# Therapeutic inhibition of miR-802 protects against obesity through AMPK-mediated regulation of hepatic lipid metabolism

**DOI:** 10.7150/thno.49354

**Published:** 2021-01-01

**Authors:** Yangyue Ni, Zhipeng Xu, Chen Li, Yuxiao Zhu, Ran Liu, Fan Zhang, Hao Chang, Maining Li, Liang Sheng, Zhong Li, Min Hou, Lin Chen, Hong You, Donald P. McManus, Wei Hu, Yinong Duan, Yu Liu, Minjun Ji

**Affiliations:** 1 Department of Pathogen Biology, Jiangsu Province Key Laboratory of Modern Pathogen Biology, Center for global health, Nanjing Medical University, Nanjing, Jiangsu, China.; 2 Department of endocrinology, the Affiliated Sir Run Run Hospital of Nanjing Medical University, Nanjing, Jiangsu, China.; 3 Key Laboratory of Rare Metabolic Disease, Nanjing Medical University, Nanjing, Jiangsu, China.; 4 Molecular Parasitology Laboratory, QIMR Berghofer Medical Research Institute, Queensland, Australia.; 5 State Key Laboratory of Genetic Engineering and Ministry of Education Key Laboratory of Contemporary Anthropology, Collaborative Innovation Center for Genetics and Development, School of Life Sciences, Fudan University, Shanghai, China.; 6 Department of Pathogen Biology, Nantong University. Nantong, Jiangsu, China.

**Keywords:** miR-802, Sjp40, *Schistosoma japonicum*, AMPK, lipid metabolism

## Abstract

**Background:** The host-parasite relationship is based on subtle interplay between parasite survival strategies and host defense mechanisms. It is well known that helminth infection, which afflicts more than one billion people globally, correlates with a decreased prevalence of obesity. Dissecting the underlying mechanisms can provide new targets for treating obesity from the host-parasite interaction perspective.

**Methods:** C57BL/6 mice received a normal or high-fat diet (HFD) with or without Sjp40 (one main component of schistosome-derived soluble egg antigens) treatment. Both the loss and gain-of-function experiments by the inhibitor suppression and lentivirus treatment of miR-802 were utilized to elucidate the role of miR-802/AMPK axis in host lipid metabolism. Hepatocyte lipogenesis assay and metabolic parameters were assessed both *in vivo* and *in vitro*. The potential interactions among Sjp40, CD36, miR-802, Prkab1, and AMPK were clarified by pull-down, miRNA expression microarray, quantitative RT-PCR, dual-luciferase reporter assay, and western blotting analysis.

**Results:** We showed a link between decreased miR-802 and impaired lipid metabolism in *Schistosoma japonicum* infected mice. The decreased miR-802 promotes murine *Prkab1* or human* Prkaa1* expression, respectively, which increases levels of phosphorylated AMPK, resulting in a decrease in hepatic lipogenesis. Also, injection with schistosome-derived soluble egg antigens (SEA) attenuated metabolism. We demonstrated that Sjp40 as a main component of SEA interacted with CD36 on hepatocytes to inhibit miR-802, resulting in the activation of AMPK pathway and subsequent attenuation of lipogenesis.

**Collectively:** Our study reveals the significant role of miR-802/AMPK axis in hepatic lipid metabolism and identifies the therapeutic potential of Sjp40 in treating obesity-related fatty liver.

## Introduction

Obesity, which is one of the most important causes of fatty liver disease worldwide, represents a growing threat to public health, not only in industrialized countries but also in urban centers of developing countries 1. Epidemiologic evidence is emerging that parasitic helminths, which infect at least one-third of the global population 2, might be associated with the considerably lower reported prevalence of obesity in developing countries 3. Consistent with this, several studies with animal models showed that helminth worm infection protects against obesity and improves insulin sensitivity 4. These studies strongly evoked our interest in identifying the mechanisms involved and to target parasites-derived molecules for treating obesity-related fatty liver disease.

Recent reports have indicated an association between microRNAs (miRNAs) and energy metabolism in mammals; these include miR-27a 5, miR-215a-5p 6, miR-33a/b 7, miR-23a/b 8, and miR-378 9. Maintaining the cellular balance of miRNAs is necessary for metabolic homeostasis as its aberrant expression is associated with an increased inflammatory response and the development of insulin resistance 10. Importantly, genome-wide association analysis has confirmed a significant association of some miRNAs with the levels of key proteins involved in cholesterol-lipoprotein trafficking, such as the ATP-binding cassette A1 (ABCA1) cholesterol transporter, further supporting the concept that miRNAs may contribute to the development of obesity and insulin resistance 11.

AMP-activated protein kinase (AMPK), which comprises a catalytic subunit (α) and two regulatory subunits (β and γ), is a crucial sensor related to host cellular energy metabolism 12. AMPK activation enhances catabolism and inhibits anabolism through the phosphorylation of acetyl-CoA carboxylase (ACC) to equilibrate energy status 13. In this study, we used the various murine models with the blood fluke *Schistosoma japonicum* (*S. japonicum*) infection or egg antigens injection to demonstrate that parasite-derived antigens (soluble egg antigens, SEA or one major component Sjp40) can downregulate hepatic miR-802 expression, resulting in up-regulation of its target gene, murine *Prkab1* (AMPKβ1) or human *Prkaa1* (AMPKα1), and subsequently alteration of hepatic lipogenesis by activation of the AMPK signaling pathway. Our studies not only shed light on some of the underlying relationships between helminth infection and host metabolism, but also reveal schistosome-derived Sjp40 as a potentially important candidate in control of lipid formation and obesity resistance through miR-802/AMPK regulation.

## Results

### *S. japonicum* infection improves lipid metabolism in mice fed with a high-fat diet

To investigate the effect of schistosome infection on host lipo-metabolism, we established an *S. japonicum-*infected high-fat diet (HFD)-induced obese mouse model (Figure S1A). As shown in Figure S1B, nine weeks post *S. japonicum* infection the body weights in the infected mice fed with normal diet (ND) or HFD were significantly reduced compared to that in uninfected mice. Mice fed with the HFD exhibited significantly higher levels of cholesterol than mice fed with the ND; however, the serum levels of cholesterol and TG were dramatically decreased in HFD mice after *S. japonicum* infection (Figure S1C).

Next, we investigated hepatic lipid metabolism by analyzing hepatic diffuse vacuolation using oil red O (ORO) staining. The results suggested that HFD-fed mice infected with *S. japonicum* accumulated fewer lipids in their livers compared with uninfected HFD-fed animals (Figure S1D and S1E). To detect synthesis of hepatic fatty acid (FA), triglyceride (TG) as well as FA oxidation which are involved in energy homeostasis, we measured the mRNA levels of hepatic lipid metabolism-related genes in *S. japonicum-*infected mice fed with a normal or high-fat diet. The mRNA levels of lipogenesis-related genes, such as *Scd1*,* Srebp1c*, *Acc*,* Fas, Dgat1* and* CD36*, decreased in the livers of *S. japonicum-*infected ND- or HFD-fed mice compared with uninfected ND- or HFD-fed mice (Figure S1F); while the fatty acid (FA) oxidation‑related genes (Cpt1 14, Mcd 15, Lcad 16, Ucp2 17) was increased. Furthermore, the mRNA levels of lipogenesis-related genes, including *Scd1*, *Acc*, *Fas*, *Dgat2*, and* CD36*, were also consistently reduced in the epididymal adipose tissue of *S. japonicum-*infected HFD mice when compared with uninfected HFD mice (Figure S1G). Collectively, our studies suggest *S. japonicum* infection exerts protective effect against obesity-induced hepatic steatosis.

### miR-802 expression is down-regulated in response to chronic *S. japonicum* infection

**Figure 1:**
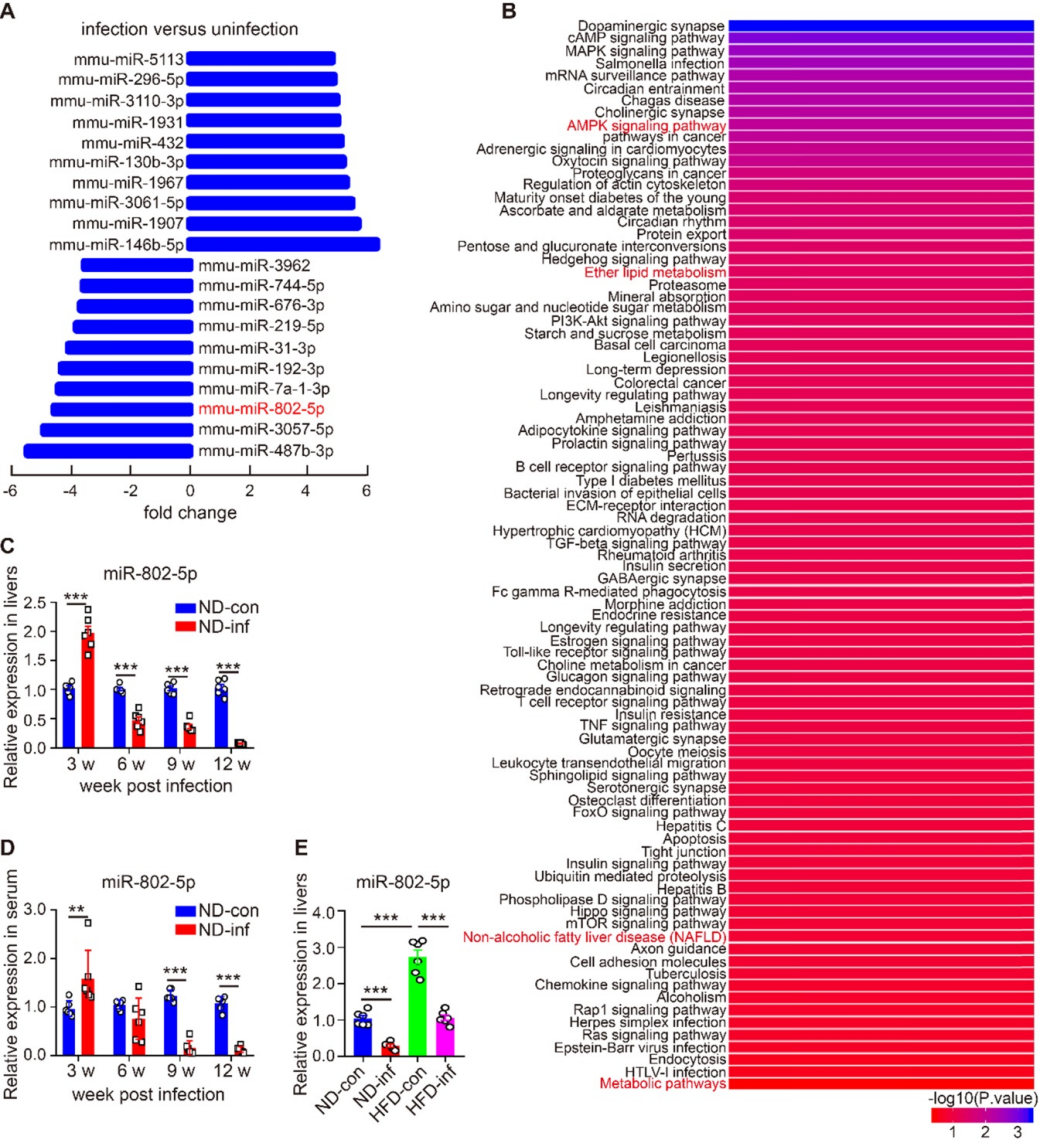
** miR-802 expression is decreased in mice infected with *S. japonicum*.** (**A-B**) Five mixed liver samples from ND-con and ND-inf mice in each group were utilized to analyze a comprehensive microRNA by the miRNA microarray chip. (**A**) 10 up-regulated and 10 down-regulated miRNAs in the liver miRNA microarray chip were selected. (**B**) Enriched signaling pathways of the top 40 target genes of miR-802. (**C**) miR-802 expression by qRT-PCR in livers of ND-con mice at 0w, 3w, 6w, 9w, and 12w after *S. japonicum* infection. (**D**) miR-802 expression by qRT-PCR in sera of C57BL/6 mice at different time-points post infection. (**E**) qRT-PCR quantification of miR-802 expression in the livers of uninfected and infected C57BL/6 mice fed with a normal chow diet and high fat diet. Data are expressed as the mean ± s.e.m of 6 mice for each group in one representative experiment. All experiments were repeated twice, *P <0.05, **P <0.01, ***P <0.001.

miRNAs modulate transcriptional and translational programs to orchestrate both physiological and pathological processes in parasite infection. Using miRNA microarray analysis, we characterized miRNA profile in normal and *S. japonicum-*infected mice. The top 10 upregulated and down-regulated miRNAs were detected in the livers of *S. japonicum-*infected mice (Figure 1A and Table S1). To determine the potential roles of these abundant miRNAs in *S. japonicum-*infected mice, we predicted the top 40 target genes of each miRNA using the Targetscan website (http://www.targetscan.org/vert_71/) and enriched their signaling pathways through the KOBAS 3.0 website (http://kobas.cbi.pku.edu.cn/anno_iden.php). We found that the target genes of miR-802-5p (miR-802-5p in this article was represented by miR-802) were the mostly highly involved in metabolic pathways such as the AMPK signaling pathway or in lipid metabolism (Figure 1B), suggesting a potential role for miR-802 in the regulation of hepatic lipid metabolism during* S. japonicum* infection.

We then used quantitative RT-PCR (qRT-PCR) to assess the dynamic expression of miR-802 in the liver and sera of mice during *S. japonicum* infection; hepatic miR-802 expression was increased in the early phase of infection (at 3 weeks post-infection), but rapidly decreased at 6, 9, and 12 weeks post infection, at the time when *S. japonicum* eggs were being deposited in the liver (Figure 1C and Figure S2). Consistent with these results, serum miR-802 expression was also significantly decreased at 9 and 12 weeks post infection (Figure 1D). In addition, obese led to enhanced expression of miR-802 in the livers of mice, while miR-802 expression was significantly decreased when HFD-fed mice were infected with* S. japonicum* (Figure 1E). Taken together, *S. japonicum* infection dramatically reduced hepatic expression of miR-802, suggesting its potentially regulatory role in hepatic lipid accumulation.

### miR-802 promotes lipogenesis through inhibiting the AMPK pathway *in vitro*

**Figure 2:**
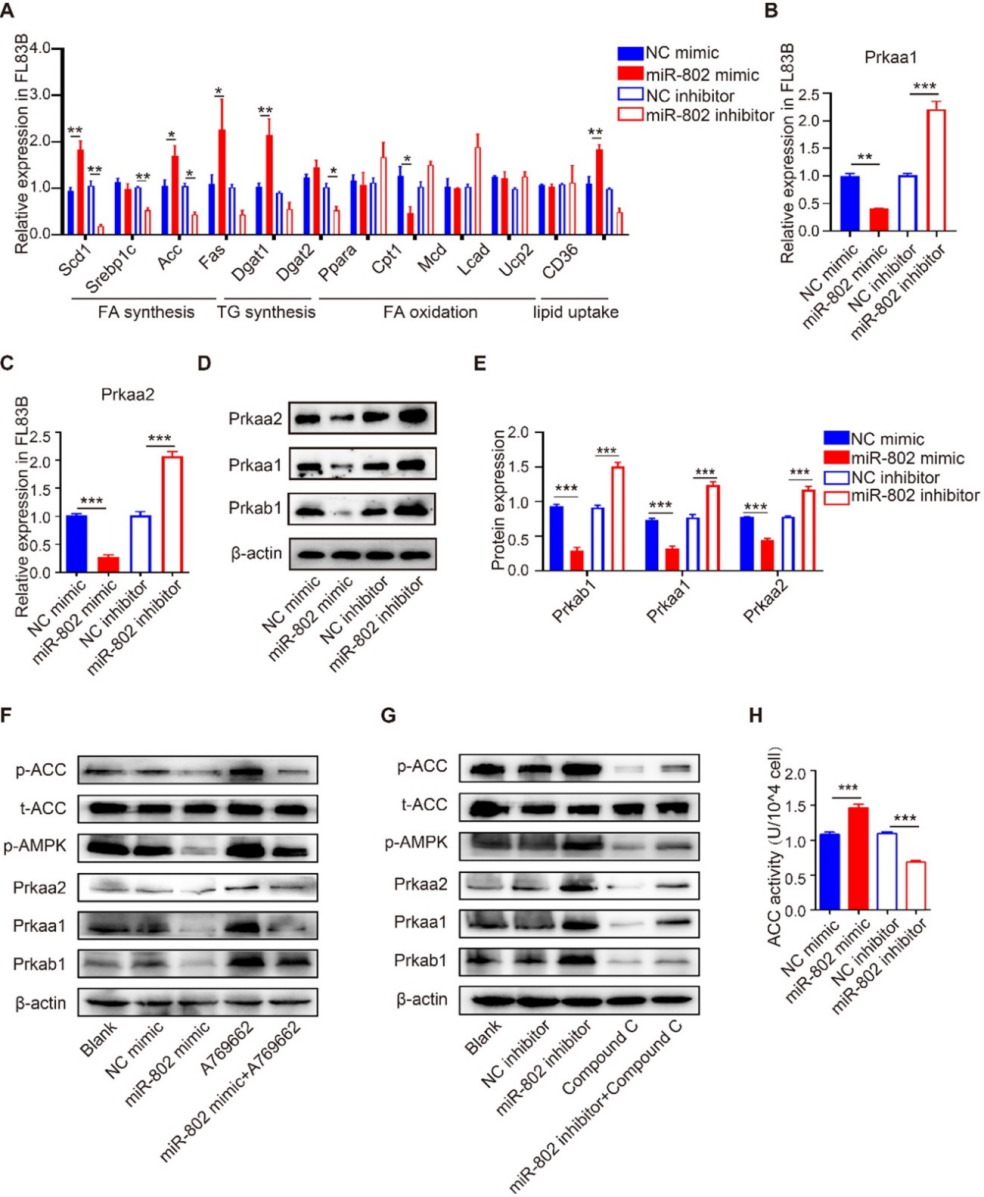
** The influence of miR-802 expression on lipid metabolism in murine hepatocytes is AMPK dependent.** (**A**) qRT-PCR analysis of gene expression related to lipo-metabolism in FL83B cells with miR-802 mimic or inhibitor treatment. (**B, C**) qRT-PCR analysis of* Prkaa1* and *Prkaa2* expression in FL83B cells upon transfection with miR-802 mimic or inhibitor. (**D, E**) Western blot analysis of protein levels of Prkab1, Prkaa1, Prkaa2 upon transfection with miR-802 mimic or inhibitor in FL83B cells. (**F**) Western blot analysis of phosphorylated AMPK and ACC in FL83B cells after stimulating with A769662, miR-802 mimic or A769662 + miR-802 mimic. (**G**) Western blot analysis of phosphorylated AMPK and ACC in FL83B cells after stimulating with miR-802 inhibitor, compound C or miR-802 inhibitor + compound C. (**H**) ACC activity in FL83B cells after treating with miR-802 mimic or inhibitor. Data are expressed as the mean ± s.e.m of 3 independent experiments with similar results. *P <0.05, **P <0.01, ***P <0.001.

To investigate the role of miR-802 in hepatic lipo-metabolism, we examined the mRNA expression level in the genes related to lipid synthesis and lipolysis by transfecting FL83B cells (a hepatocyte cell line) with miR-802 mimic or miR-802 inhibitor. The results showed that miR-802 obviously increased the expression of *Scd1*, *Acc*, *Fas*, *Dgat1* and* CD36*, but reduced the expression of *Cpt1* in FL83B cells. In contrast, miR-802 inhibitor reduced the expression of *Scd1*, *Srebp1c*, *Acc*, *Dgat2* and *CD36* were significantly decreased (Figure 2A).

Prkab1 (β subunit of AMPK, AMPKβ1) is a predicted murine target gene of miR-802. It is noteworthy that AMPKβ1 is a well-known bioenergetics sensor (Table S2), critical for maintenance of energetic homoeostasis through activating the α subunits (AMPKα1 & AMPKα2, also known as Prkaa1 & Prkaa2, respectively) 18, 19; we hypothesized that miR-802 regulates Prkab1 expression and activates Prkaa1 and Prkaa2 in hepatocytes. Our results showed the mRNAs and proteins levels of Prkab1, Prkaa1 and Prkaa2 were significant decreased after miR-802 mimic treatment in FL83B cells, while they were increased in miR-802 inhibitor-treated FL83B cells (Figure 2B-2E).

AMPK reduces lipogenesis through phosphorylation of its downstream substrate ACC, a process that inhibits the expression of lipid synthesis-related transcription factors 20, 21. Consistently, the levels of phosphorylated AMPK and ACC were enhanced in FL83B cells that were pretreated with AMPK activator A769662, whereas administration of a miR-802 mimic reversed this upregulation (Figure 2F). Importantly, we found that transfection of miR-802 inhibitor substantially increased phosphorylated AMPK (Thr172) and subsequent phosphorylated ACC, whereas compound C, an inhibitor of AMPK, attenuated the activation of AMPK and the phosphorylation of ACC in FL83B cells (Figure 2G). Similar results were obtained with another inhibitor of AMPK, SBI-0206965 (Figure S3G). Furthermore, overexpression of miR-802 significantly upregulated ACC activity, while suppression of miR-802 reduced ACC activity in FL83B cells (Figure 2H).

miR-802-mediated effect on lipo-metabolism were also detected in murine primary hepatocytes (MPHs). miR-802 overexpression in MPHs resulted in reduced expression of Prkaa1 and Prkaa2, and vice versa (Figure S3A-S3D). Importantly, the incorporation of ^3^H-glucose into fatty acids was enhanced in miR-802 mimic-stimulated MPHs (Figure S3E). Together, these observations further suggested that miR-802 is a critical activator of hepatic lipogenesis by its interaction with the AMPK pathway.

### miR-802 was confirmed to bind* Prkab1* and regulate its expression

**Figure 3:**
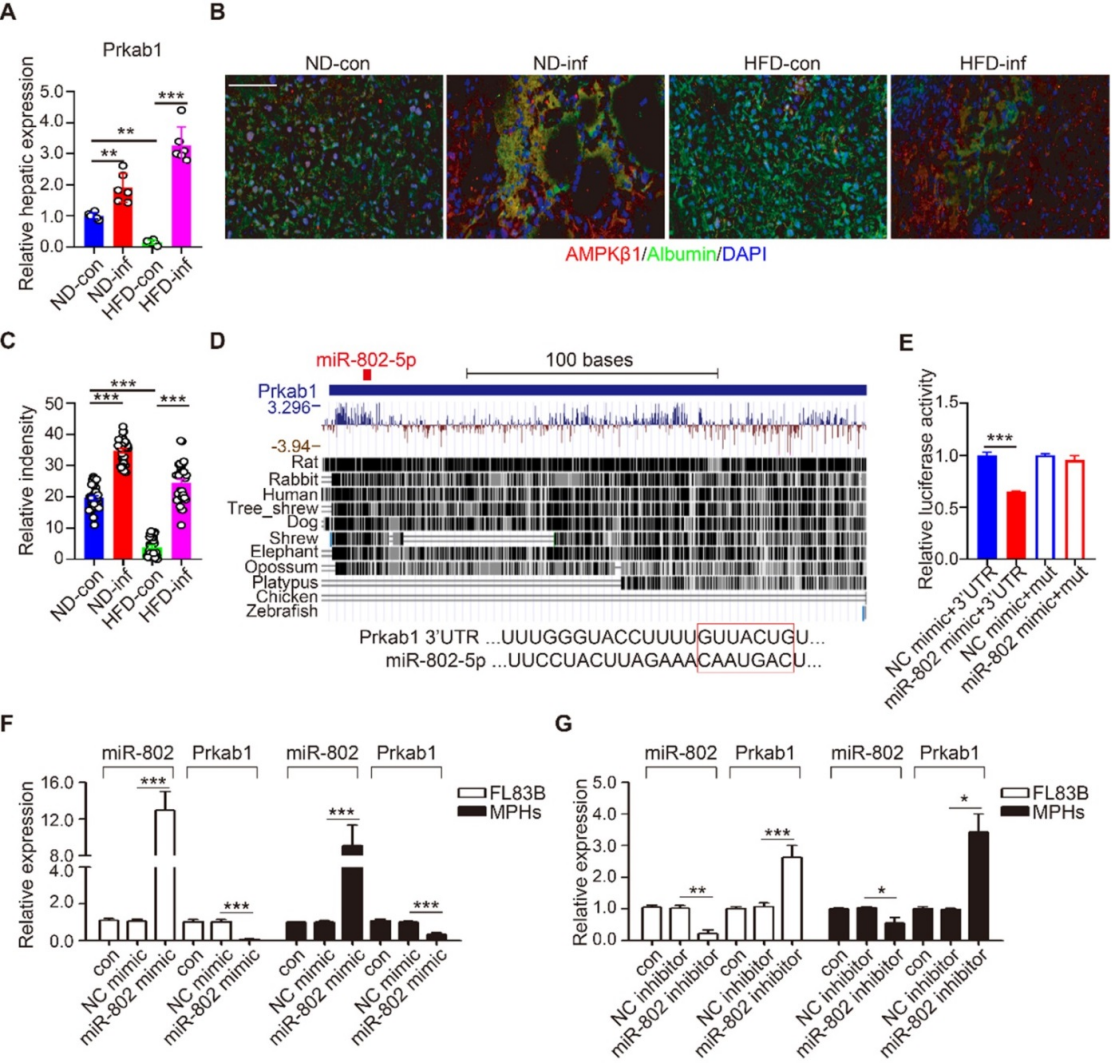
** miR-802 regulates the expression of* Prkab1*.** (**A**) qRT-PCR quantification of *Prkab1* expression in livers of uninfected and infected C57BL/6 mice fed with ND and HFD. (B) Representative figures of Prkab1/Albumin double immunostaining on liver sections of four groups of mice. (**C**) Relative intensity data of Prkab1/Albumin double immunostaining cells (5 random liver fields in each mouse, n = 6 mice per group). (**D**) Conservation of miR-802 target regions in the 3'UTR of *Prkab1*. (**E**) Luciferase report assays for 293T cells transfected with pMIR-Report Luciferase vectors carrying wild type (WT) or mutated (MuT) 3'UTR of *Prkab1* in the presence of miR-802 mimic or NC-mimic. (**F**) qRT-PCR quantification of miR-802 and *Prkab1* expression in FL83B cells and MPHs treated with miR-802 mimic. (**G**) qRT-PCR quantification of miR-802 and *Prkab1* expression in FL83B cells and MPHs when using miR-802 inhibitor. Data are expressed as the mean ± s.e.m of 3 (n = 3 in E, F, G) or 2 (n = 6 in A, B, C) repeated experiments. *P <0.05, **P <0.01, ***P <0.001.

Prkab1, Ppp2ca, and Pafah1b1 have potential roles in the upregulation of lipogenesis. Using qRT-PCR, we demonstrated that *S. japonicum* infection significantly increased expression of *Prkab1*, but not *Ppp2ca*, and *Pafah1b1* in the livers of ND-fed mice compared with that in the control mice with infection (Figure 3A and Figure S4A). HFD-induced obese significantly reduced hepatic mRNA level of *Prkab1*, which can be released by *S. japonicum* infection (Figure 3A). We determined the protein expression of Prkab1 by immunofluorescence staining which revealed that the location of Prkab1 (red) in the cytoplasm of hepatocytes (green). The protein of Prkab1 dramatically increased in the livers from ND- or HFD-fed mice with *S. japonicum* infection. (Figure 3B, 3C).

miRNAs are able to potentially bind many mRNAs to affect their expression and stability. Using Targetscan analysis, we found that *Prkab1* 3'-UTR harbors an evolutionarily conserved binding site for miR-802 (Figure 3D) 22, which display 100% complementary to the murine miR-802 5' conserved seed region with the highest prediction score. To test whether the binding sites were functional, we co-transfected 293T cells with a luciferase reporter plasmid containing *Prkab1* 3'-UTR, miR-802 mimics, or scramble mimics (NC). The results showed that overexpression of miR-802 resulted in suppression of luciferase intensity in cells transfected with *Prkab1* containing 3'-UTR, this effect was not observed in the cells stimulated with mutant 3'-UTR (Figure 3E). There results indicate a direct interaction between miR-802 and *Prkab1* mRNA.

Next, we investigated if miR-802 regulates *Prkab1* expression in hepatocytes. miR-802 mimic or inhibitor were transfected into FL83B and MPHs, respectively. miR-802 transfection reduced *Prkab1* expression in both cells (Figure 3F), but miR-802 inhibitor enhanced *Prkab1* expression (Figure 3G). In addition, miR-802 mimic-treated MPHs did not significantly affect the expression of *Ppp2ca* and *Pafah1b1* (Figure S4B), further suggesting that the selection of *Prkab1* as a target gene of miR-802 was appropriate. Overall, these data indicate that miR-802 directly targets* Prkab1* and suppresses its expression.

**Prkab1 activates AMPK to reduce lipid production in hepatocytes**

**Figure 4:**
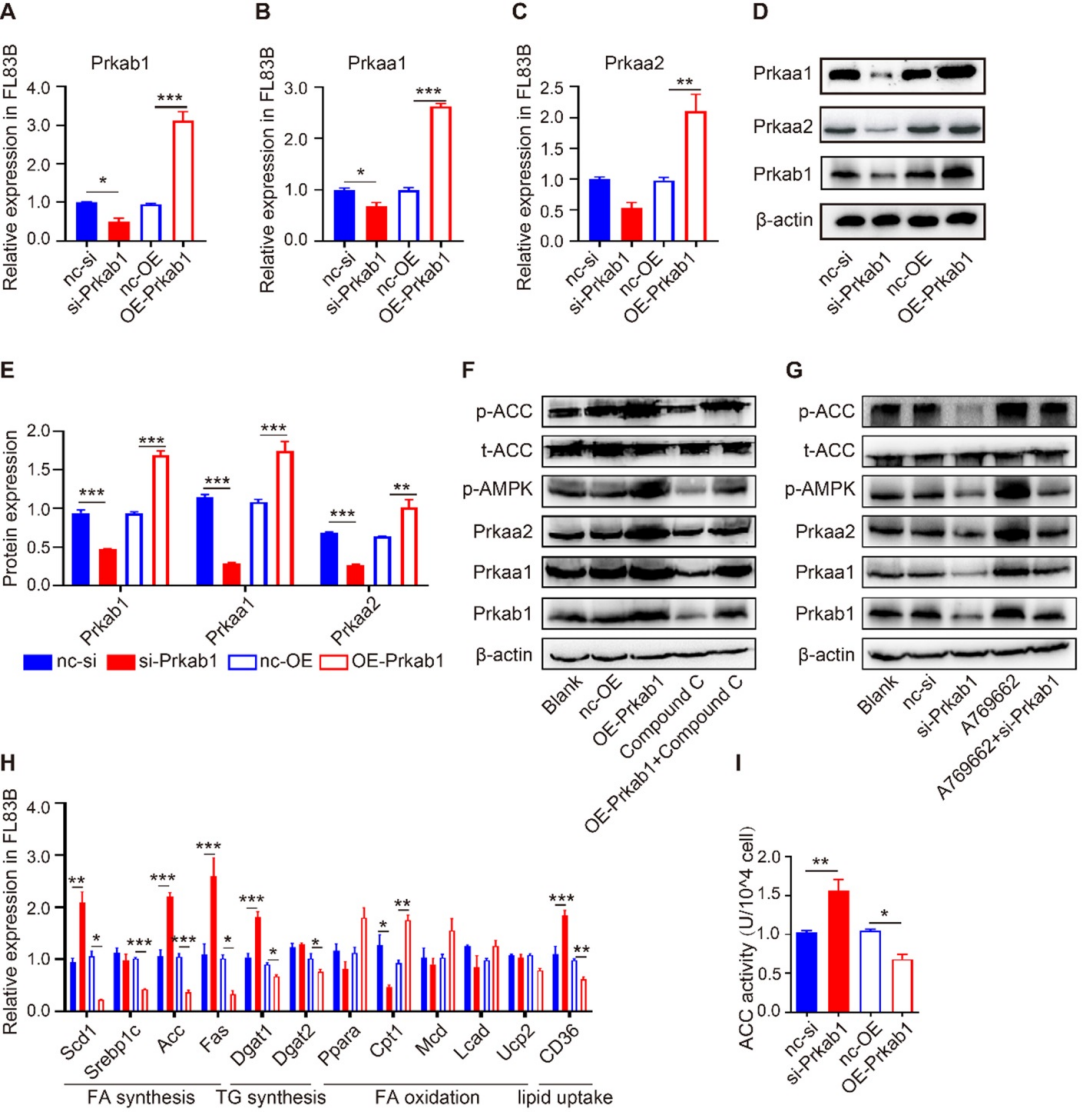
** Prkab1 activated AMPK to promote the oxidation and suppress the synthesis of fatty acid.** (**A-C**) qRT-PCR analysis of *Prkab1*, *Prkaa1* and *Prkaa2* expression in FL83B cells upon transfection with si-*Prkab1* or OE-*Prkab1*. (**D, E**) Western blot analysis of protein levels of Prkab1, Prkaa1 and Prkaa2 in FL83B cells after transfecting with si-*Prkab1* or OE-*Prkab1*. (**F**) Western blot analysis of phosphorylated AMPK and ACC in FL83B cells with OE-*Prkab1*, compound C or OE-*Prkab1* + compound C treatment. (**G**) Western blot analysis of phosphorylated AMPK and ACC in FL83B cells with A769662, si-*Prkab1* or A769662 + si-*Prkab1* treatment. (**H**) qRT-PCR analysis of lipogenesis-related genes expression in FL83B cells when transfected with si-*Prkab1* or OE-*Prkab1*. (**I**) ACC activity assay of FL83B cells upon treatment of si-*Prkab1* or OE-*Prkab1.* Error bars represented mean ± s.e.m of 3 independent repeat experiments. *P <0.05, **P <0.01, ***P <0.001.

To assess if Prkab1 regulates hepatic lipogenesis through modulating AMPK, hepatocytes were transfected with OE-*Prkab1* plasmid or si-*Prkab1* to provide overexpression or knockdown of* Prkab1*, respectively*.* Oil red staining revealed that si-Prkab1 treatment led to significantly higher levels of intracellular lipids in FL83B cells (Figure S5F). The results showed that overexpression of *Prkab1* significantly increased the mRNA and protein levels of Prkaa1 and Prkaa2, while knockdown of *Prkab1* decreased the expression of Prkaa1 and Prkaa2 in FL83B cells (Figure 4A-4E). In addition, the levels of phosphorylated AMPK and ACC were significantly increased after overexpression of* Prkab1*; however, treatment with compound C, an inhibitor of AMPK, inhibited *Prkab1*-mediated AMPK activation (Figure 4F). Furthermore, A769662, an AMPK activator, increased the phosphorylation of AMPK and ACC, while knockdown of *Prkab1* weakened this effect in A769662-treated FL83B cells (Figure 4G), suggesting *Prkab1* is essential for the activation of the AMPK pathway. Similar results were also observed in murine primary hepatocytes (MPHs) cells (Figure S5).

To determine the contribution of *Prkab1* to the altered hepatic lipid metabolism, we demonstrated that overexpression of *Prkab1* in FL83B cells resulted in reduced mRNA expression of *Scd1*, *Srebp1c*, *Acc*, *Fas*, *Dgat1*, *Dgat2* and *CD36*, but increased mRNA levels of fatty acid (FA) oxidation‑related gene *Cpt1* (Figure 4H). Consistently, we found that up-regulation of *Prkab1* decreased ACC activity through phosphorylation of ACC, suggesting that AMPK activation can inhibit ACC activity (Figure 4I). Together, the increased *Prkab1* expression appeared to elicit activation of the AMPK pathway and reduce lipogenesis-related gene expression in the host liver.

### miR-802 is essential for lipid accumulation during *S. japonicum* infection

**Figure 5:**
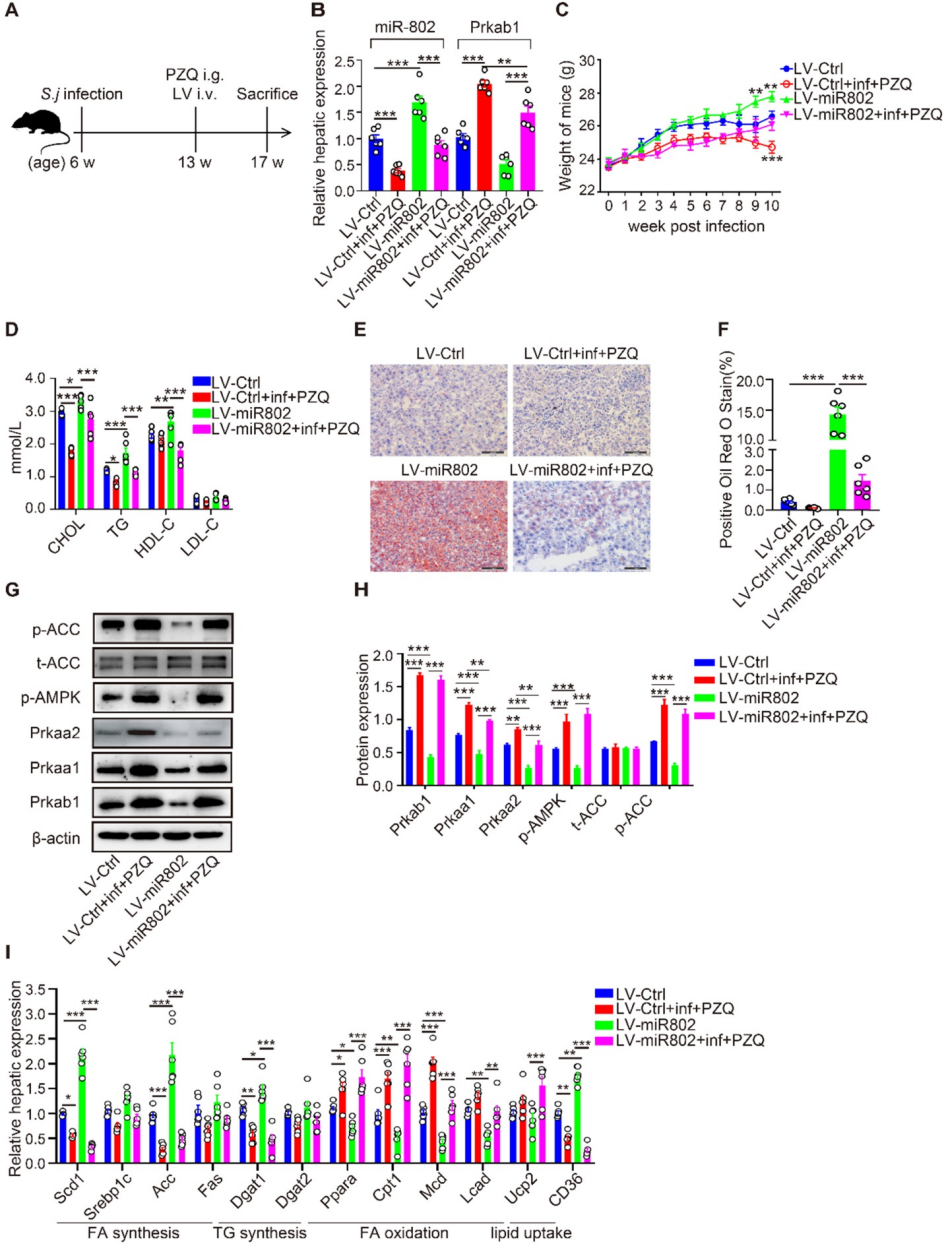
** miR-802 overexpression leads to host lipid accumulation, which can be attenuated by *Schistosoma japonicum* infection.** (**A**) Mice were randomly divided into four groups: Lv-Ctrl, Lv-miR802, LV-Ctrl+inf+PZQ, and LV-miR802+inf+PZQ groups. Each mouse in the four groups was intravenously administered 5×10^7^ TU lentivirus (LV-Ctrl) or lentivirus-miR-802 (LV-miR802) in 200 µl PBS once a week continuously for four weeks. Data are indicated mean ± s.e.m of 6 mice for each group in one representative experiment; all experiments were repeated twice. *P < 0.05, **P < 0.01, ***P < 0.001. (**B**) Expression of miR-802 and *Prkab1* in the livers of mice from these groups. (**C**) Dynamic changes in body weight of mice in the LV-Ctrl, LV-miR802, LV-Ctrl+inf+PZQ, and LV-miR802+inf+PZQ groups. (**D**) Cholesterol, TG, HDL-C, LDL-C levels in the sera of the four groups of mice. (**E, F**) Representative images of liver sections stained with Oil red. (**G, H**) The expression of Prkab1, Prkaa1, Prkaa2, phosphorylated AMPK, total ACC, and phosphorylated ACC in the livers from mice in each group. Data are expressed as the mean ± s.e.m. from each group, and are representative of one typical experiment out of three, *P < 0.05, **P < 0.01, ***P < 0.001. (I) qRT-PCR quantification of the expression of lipogenesis-related genes in the livers of mice from each group.

Next, we assessed the influence of reduced miR-802 on the regulation of lipid metabolism during *S. japonicum* infection. In order to avoid the interference of excessive inflammatory reactions after* S. japonicum* infection, *S. japonicum-*infected mice received the treatment with praziquantel (PZQ) (Figure 5A, S6A and S6B), as this treatment has been reported to prevent strong inflammation while maintaining a low level of host metabolism 23, making it suitable for study following intravenous injection with miR-802 lentivirus (LV). LV-miR-802 restores miR-802 levels in the livers, adipose, pancreas and skeletal muscle of *Schistosoma japonicum* mice (Figure 5B, S6C, S6D). We found that upregulation of miR-802 resulted in significant decrease in the expression of* Prkab1* both in the livers (Figure 5B) and adipose tissues (Figure S6D) in LV-miR-802-injected mice compared with control animals. In addition, the body weights of the LV-miR-802 or LV-miR-802+inf+PZQ groups exhibited a marked increase when compared with the LV-Ctrl or LV-Ctrl+inf+PZQ groups, respectively (Figure 5C). Although the schistosome infection decreased the serum TG and cholesterol levels, overexpression of miR-802 significantly upregulated the serum TG and cholesterol levels but reduced serum HDL-C levels in mice (Figure 5D).

Furthermore, *S. japonicum* infected mice receiving PZQ therapy had a reduced number of lipid droplets in the liver, with increased levels of phosphorylated AMPK and ACC. However, LV-miR-802 injection promoted hepatic lipid accumulation in liver tissue (Figure 5E, 5F), as well as a decrease in phosphorylated AMPK and ACC levels (Figure 5G, 5H). Additionally, the expression of genes related to fatty acid and triglyceride synthesis (*Scd1*, *Acc*,* Dagt1*and CD36) was decreased in the PZQ-treated *S. japonicum-*infected group, while these genes were upregulated after injection with LV-miR-802 (Figure 5I). Moreover, the expression of *Scd1*, *Srebp1c*, *Acc*, *Fas*, *Dgat1*, *Dgat2* and *CD36* was also decreased in the epididymal adipose tissue when schistosome infection, but was increased when administration with LV-miR802 (Figure S6E). Collectively, these findings show that miR-802 is essential for the deposition of lipid in the liver.

### SEA suppresses miR-802 expression and improves lipid metabolism

To gain insights into the signaling events linked to dysregulated miRNA-802 expression, we began by identifying the upstream regulators of miRNA-802. We focused on *S. japonicum* eggs because of their ability to secrete abundant antigenic molecules 24. Using transwell culture system, we detected the reduced expression of miR-802 and increased *Prkab1* in murine primary hepatocytes (MPHs) after receiving stimulation with egg-secreted proteins (ESPs) (Figure S7A) or SEA (Figure S8A), suggesting that egg-derived proteins may contribute to the dysregulation of miR-802 in the hepatocytes. Then, FL83B cells were stimulated with SEA for 6, 12, 24, and 48 hours and the results showed that miR-802 transcription was significantly decreased since 6 hours post treatment, which was more rapid than the increased expression of* Prkab1* (Figure S7B, S7C). Consistent with *in vitro* data,* in vivo* intraperitoneal injection of SEA significantly decreased the expression of miR-802 in the livers of ND- or HFD-fed mice when compared with saline-only treated mice. Also, the hepatic mRNA level of *Prkab1* was enhanced after SEA injection (Figure S8B). Together, these observations suggest that SEA have certain potential capability to function upstream of miR-802 and regulate *Prkab1* expression. To assess the effect of SEA on lipid metabolism in hepatocytes, we examined the mRNA levels of metabolic genes in SEA-treated FL83B cells. The result showed that the expression of lipogenesis-related genes (*Dgat1* and* CD36*) was suppressed, but the expression of genes related to fatty acid oxidation (*Cpt1*, *Mcd*, and *Ucp2*) was increased under SEA stimulation (Figure S8C). In addition, SEA increased the protein level of Prkab1, while Prkab1 was partially suppressed with SEA and miR-802 mimic together (Figure S7D).

To further investigate the role of SEA in the regulation of hepatic lipogenesis* in vivo,* HFD-fed mice were treated with SEA (Figure S8D). Body weight loss were detected in both ND-fed and HFD-fed mice after receiving injection of SEA compared with the controls injected with saline (Figure S8E). Consistent with these results, SEA treatment decreased the circulating levels of cholesterol, triglyceride, and LDL-C in ND-fed mice and HFD-fed mice (Figure S8F). Additionally, SEA injection resulted in reduced levels of lipid droplets in the liver in HFD-fed mice (Figure S8G, S8H). Furthermore, the protein levels of Prkab1, Prkaa1, Prkaa2, phosphorylated AMPK, and phosphorylated ACC in the livers of HFD-fed mice were reduced when compared with ND-fed mice, whereas SEA-treated ND-fed and HFD-fed mice showed a clear increase in the levels of Prkab1, Prkaa1, Prkaa2, phosphorylated AMPK, and phosphorylated ACC when compared to the corresponding control mice (Figure S8I, S8J). Consistent with the data from SEA-injected mouse model, similar results were observed in the livers of ND-fed and HFD-fed mice after *S. japonicum* infection (Figure S1H, S1I). As a consequence, essential FA synthesis genes such as *Scd1*, *Srebp1c*, *Acc* and *Fas* were decreased, while the expression of genes required for FA oxidation (such as *Cpt1*) showed a marked increase in the livers of SEA-treated ND- or HFD-induced obese mice (Figure S8K). Thus, the SEA could activate AMPK pathway via attenuating miR-802 expression to participate in regulating hepatic lipid metabolism in obese mice.

### Sjp40 improves lipid metabolism in HFD-induced obese mice

The composition of SEA is complex and diverse, and a deeper understanding of specific egg-derived molecular components could lead to improved resolution of metabolic disease. We prepared HSP40 protein (Sjp40), a major secreted constituent of *S. japonicum* SEA (22, 23) (Figure S9A) and stimulated the hepatocytes with purified Sjp40. We found that *in vitro* treatment with Sjp40 led to significantly decrease of intracellular lipids (Figure S9H), which further down-regulated expression of miR-802 and increased expression of *Prkab1* in FL83B cells, or in MPHs cells (Figure S9B-9D). Accordingly, Sjp40-stimulated FL83B and MPHs cells showed the similar expression characteristics with lower expression of genes related to FA and TG synthesis and higher expression of genes associated with FA oxidation (Figure S9E, S9F). The incorporation of ^3^H-glucose into fatty acids was significantly decreased in SEA- or Sjp40-stimulated murine primary hepatocytes (Figure S9G).

**Figure 6:**
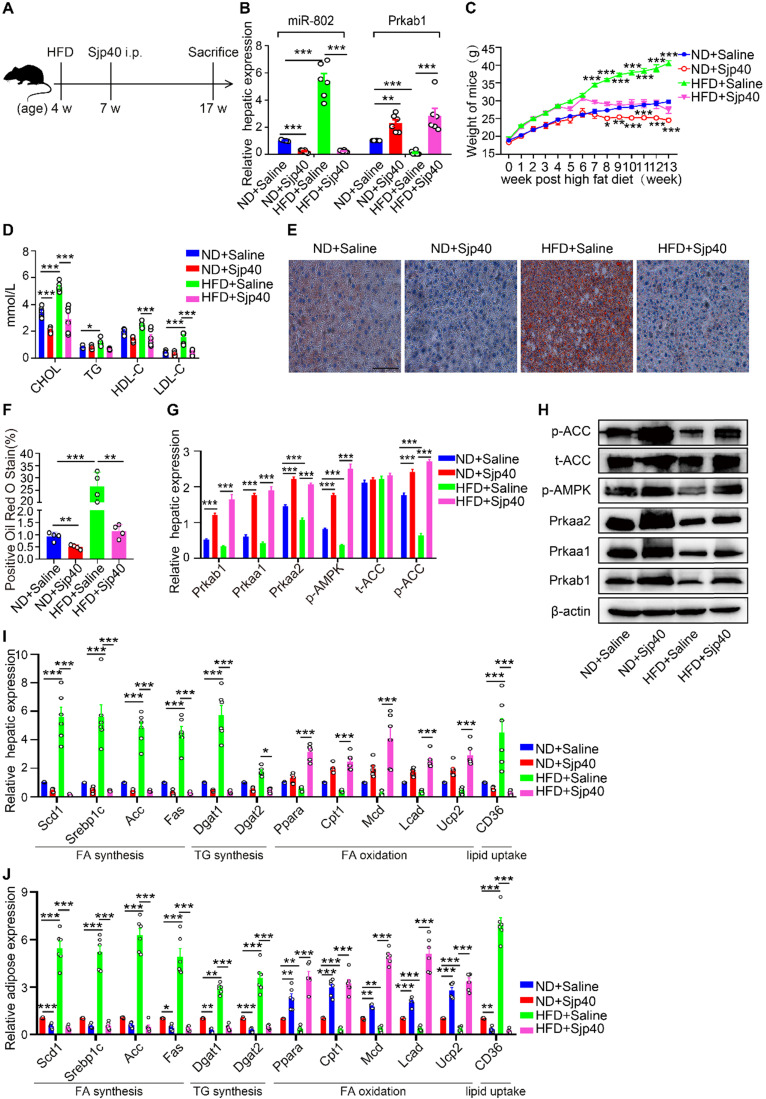
** Sjp40 improves lipid metabolism in HFD mice.** (**A**) Mice were randomly divided into four groups: the ND-Saline, ND-Sjp40, HFD-Saline and HFD-Sjp40. Mice in the ND-Sjp40 and HFD-Sjp40 groups were injected *i.p*. with 50 µg Sjp40 twice a week for 10 weeks. (**B**) Expression of miR-802 and *Prkab1* in the livers of mice from the ND-Saline, ND-Sjp40, HFD-Saline group or HFD-Sjp40 group. (**C**) Dynamic changes in body weight of mice in the ND-Saline, ND-Sjp40, HFD-Saline and HFD-Sjp40 groups. (**D**) Cholesterol, TG, HDL-C, LDL-C levels in the sera of ND-Saline, ND-Sjp40, HFD-Saline and HFD-Sjp40 mice. (**E, F**) Representative images of liver sections stained with Oil red O (Scale bars=100 µm). (**G, H**) Detection of Prkab1, Prkaa1, Prkaa2, phosphorylated AMPK, total ACC and phosphorylated ACC levels in livers of these four groups of mice. Data are expressed as the mean ± s.e.m. from each group, and are representative of one typical experiment out of three, ***P<0.001, **P<0.01. (**I**) qRT-PCR quantification of the expression of lipogenesis-related genes in the livers of these four groups of mice. (**J**) qRT-PCR quantification of lipogenesis-related genes expression in the adiposes of mice after injecting with Sjp40. All error bars indicate mean ± s.e.m of 4 mice from each group in one representative experiment; all experiments were repeated twice. *P <0.05, **P <0.01, ***P <0.001

Furthermore, we injected Sjp40 into ND and HFD mice (Figure 6A) in order to ascertain the therapeutic efficacy of Sjp40 against obese. Consistent with *in vitro* findings, intraperitoneal injection with Sjp40 rendered attenuated miR-802 and enhanced Prkab1 expression in the livers (Figure 6B), and displayed a series of lipid-lowering effects, characterized by decreased body weight, circulating lipid levels, and reduced numbers of hepatic lipid droplets (Figure 6C-6F) without impairing food intake and water intake (Figure S10). Furthermore, the protein levels of Prkab1, Prkaa1, Prkaa2, phosphorylated AMPK, and phosphorylated ACC in the livers of ND-fed and HFD-fed mice were increased after Sjp40 treatment (Figure 6G, 6H), an outcome likely associated with the dysregulated expression of lipid metabolism-related genes both in the liver and adipose tissue (Figure 6I, 6J). Taken together, these results suggest that Sjp40, through its interaction with the miR-802/*Prkab1* axis, has potential to become a novel treatment for obesity-related fatty liver disease.

### Sjp40 binds to CD36 and inhibits miR-802 expression via NF-κB signaling

**Figure 7:**
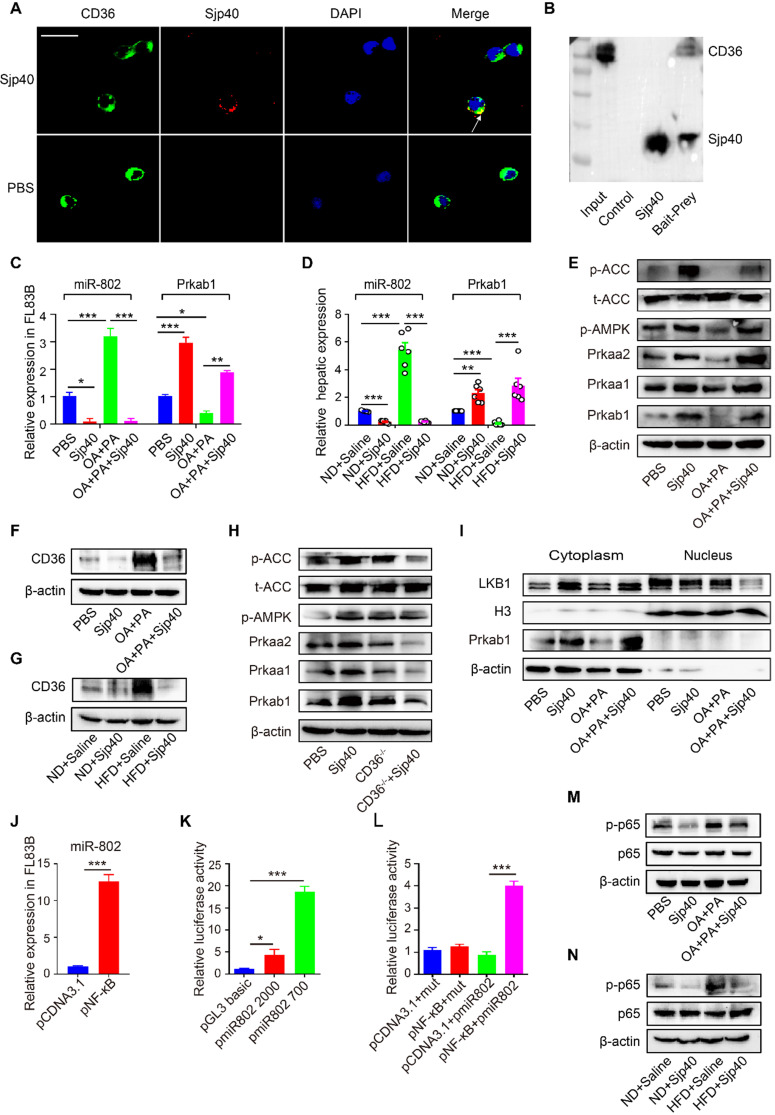
** Sjp40 inhibits miR-802 expression through suppressing CD36 and NF-κB signaling.** (**A**) After treatment with Sjp40 (10 µg/ml) for 15 min, immunoblot analysis of CD36 (green), Sjp40 (red) and DAPI (blue) expression in FL83B cells. (**B**) Pierce pull-down polyhistidine assay was employed for identifying the interaction between Sjp40 and CD36. (**C**) qRT-PCR analysis of *Prkab1* and miR-802 expression in palmitate and oleic acid-stimulated FL83B cells upon treatment with Sjp40 for 24 h. (**D**) qRT-PCR quantification of miR-802 and *Prkab1* in the livers. (**E**) Western blot analysis of palmitate and oleic acid-stimulated AMPK-ACC pathway in FL83B cells in absence or presence of Sjp40. (**F**) Western blot analysis of palmitate and oleic acid-stimulated CD36 protein levels in FL83B cells in absence or presence of Sjp40. Data are expressed as the mean ± s.e.m. for each group, and are representative of one typical experiment out of three. (**G**) Levels CD36 in the livers of four groups of mice. Data are expressed as the mean ± s.e.m. of 6 mice for each group in one representative experiment. All experiments were repeated twice. (**H**) Western blot analysis of p-AMPK and p-ACC levels in CD36^-/-^ primary hepatocytes in absence or presence of Sjp40. (**I**) Western blot analysis of LKB1 levels in FL83B cells in absence or presence of Sjp40. (**J**) qRT-PCR analysis of miR-802 in FL83B cells upon overexpression of NF-κB. (**K, L**) Luciferase activity of miR-802 promotor. 293T cells transfected with reporter constructs that contained the miR-802 promoter were treated with pNF-κB. After 48 h, luciferase activity was analyzed and plotted. Error bars represented mean ± s.e.m of 3 independent repeat experiments. (**M**) Protein Levels of p65 and p-p65 in palmitate and oleic acid-stimulated FL83B cells after stimulating with Sjp40. (**N**) Protein levels p65 and p-p65 in the livers of four groups of mice. Data are expressed as the mean ± s.e.m. for each group, and are representative of one typical experiment out of three. *P <0.05, **P <0.01, ***P <0.001.

CD36, one of the major pattern recognition receptors expressed on hepatocytes, plays a critical role in the promotion of fatty liver 25. To ascertain if Sjp40 inhibits miR-802 expression in hepatocytes through suppression of CD36 signaling, we firstly used the Protein-Protein Affinity Predictor (https://www.iitm.ac.in/bioinfo/PPA_Pred-/prediction. html) to determine whether Sjp40 could interact with CD36. The prediction result showed that there was a possibility of these two proteins interacting (Table S3). Then, we utilized confocal microscope to observe the co-localization of Sjp40 and CD36 on the membrane of hepatocytes (indicated by the yellow overlap in Figure 7A). It demonstrates that Sjp40 could interact with CD36 on hepatocytes. To further validate the interaction between Sjp40 and CD36, pull-down polyhistidine assay was performed and results showed that endogenously expressed CD36 could be enriched by His tagged Sjp40 (Figure 7B).

Next, we observed that the expression of CD36 in hepatocytes was activated with fatty acid (oleic acid and palmitic acid, OA/PA) and were suppressed with Sjp40 treatment *in vitro*. Activation of CD36 in hepatocytes resulted in increased expression of miR-802 but a decreased mRNA level of *Prkab1*; whereas treatment with Sjp40 significantly reduced expression CD36 and miR-802 but enhanced phosphorylation of AMPK pathway in OA/PA-stimulated hepatocytes (Figure 7C, 7E and 7F). Consistent with these *in vitro* observations, *in vivo* treatment of HFD-fed mice with Sjp40 also attenuated miR-802 but increased *Prkab1* expression through inhibition of CD36 (Figure 7D, 7G). After stimulating the hepatocytes from WT and CD36^-/-^ mice with Sjp40, we found that the phosphorylated AMPK and ACC were decreased in Sjp40-treated CD36^-/-^ hepatocytes when compared with Sjp40-treat WT hepatocytes, suggesting that CD36 is necessary for Sjp40-induced activating AMPK/ACC pathway (Figure 7H). Since CD36 had been reported to inhibit AMPK activation by promoting nuclear LKB1 localization 26, we found that treatment with Sjp40 resulted in decreasing the nuclear expression of LKB1 in CD36-overexpressed FL83B cells, which was activated by OA/PA (Figure 7I).

NF-κB signaling was reported to play potential role in the induction of miR-802 in keratinocytes 27, 28, we found that overexpression of NF-κB in FL83B cells promoted the expression of miR-802 (Figure 7J). We further examined the putative promoter extending 2000 bp and 700 bp upstream of the coding region of pre-miR-802 (Figure 7K), and predicted the potential binding sites of p65 in the putative mouse miR-802 promoter (http://alggen.lsi.upc.es/cgi-bin/promo_v3/promo- /promoinit.cgi?dirDB=TF_8.3). Overexpression of p65 enhanced the activity of the miR-802 promoter, while a mutation in the binding site (GGGAATGTCGG) abolished this enhancement (Figure 7L), indicating that NF-κB is important for activation of miR-802.

It was reported that CD36 can trigger NF-κB signaling. Our results showed that OA/PA stimulation in hepatocytes or HFD feeding in mice can induce upregulation of CD36 expression in the liver cells and increase phosphorylated p65 level, while Sjp40 treatment significantly attenuated the phosphorylated p65 through the downregulation of CD36 (Figure 7M and 7N). Thus, Sjp40 inhibits NF-κB signaling and reduces miR-802 expression through suppressing CD36.

### Sjp40 inhibits miR-802/*Prkaa1* axis through CD36 in human HEPG2 cells

**Figure 8:**
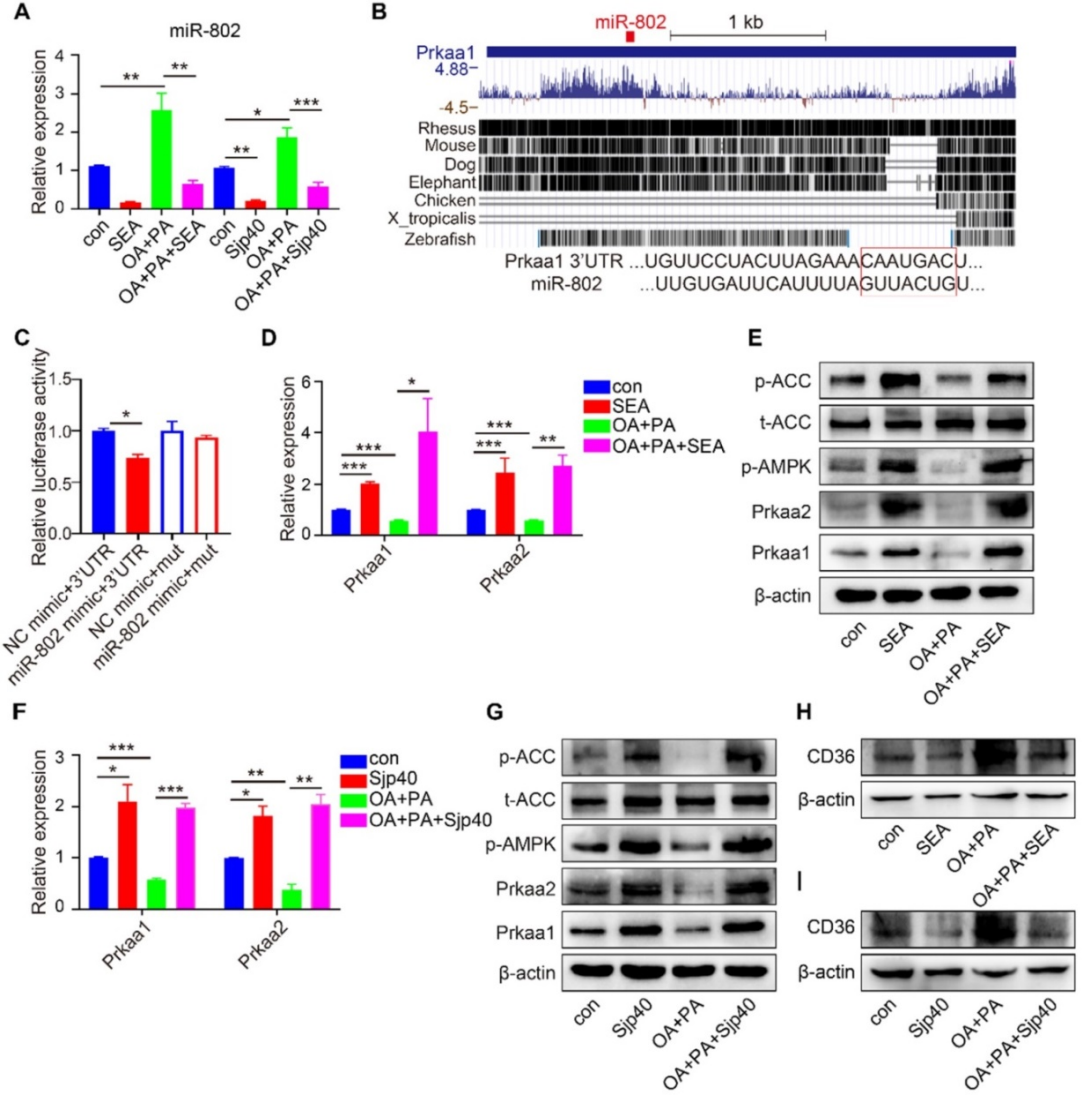
** Sjp40 activates AMPK through regulating miR-802/Prkaa1 axis in human HEPG2 cells.** (**A, D, F**) qRT-PCR analysis of miR-802, *Prkaa1* and *Prkaa2* expression in palmitate and oleic acid-stimulated HEPG2 cells upon treatment with SEA or Sjp40 (10 µg/ml) for 24 h. (**B**) Conservation of miR-802 target regions in the 3'UTR of *Prkaa1*. (**C**) Luciferase report assays for 293T cells transfected with pMIR-Report Luciferase vectors carrying wild type (WT) or mutated (MuT) 3'UTR of *Prkaa1* in the presence of miR-802 mimic or NC-mimic. (**E**) Western blot analysis of palmitate and oleic acid-stimulated AMPK and ACC protein levels in HEPG2 cells in absence or presence of SEA. (**G**) Protein levels of Prkaa1, Prkaa2, p-AMPK, ACC and p-ACC in palmitate and oleic acid-stimulated HEPG2 cells after stimulating with Sjp40. (**H**) Protein levels of CD36 in palmitate and oleic acid-stimulated HEPG2 cells after stimulating with SEA. (**I**) Western blot analysis of palmitate and oleic acid-stimulated CD36 protein levels in HEPG2 cells in absence or presence of Sjp40. Data are expressed as the mean ± s.e.m of 3 repeated experiments. *P <0.05, **P <0.01, ***P <0.001.

To better characterize the role of Sjp40 in the regulation of miR-802 in human cells, we performed RT-PCR on SEA- or Sjp40-treated human HEPG2 cell line with or without OA/PA stimulation. In accordance with data from murine hepatocytes, activation of CD36 in human HEPG2 cells resulted in increased expression of miR-802 but the decreased mRNA level of *Prkaa1* (Figure 8A, 8D, 8H and 8I), which is the potential functional target of miR-802 in human; while SEA or Sjp40 treatment reduced expression of miR-802 and CD36 (Figure 8A, 8H and 8I) but enhanced the mRNA and protein levels of* Prkaa1* or *Prkaa2* in OA/PA-treated human HEPG2 cells (Figure 8D-8G). We co-transfected 293T cells with the luciferase reporter plasmid to show that the *Prkaa1* 3'-UTR activity was suppressed after overexpression of miR-802. However, mutation of the miR-802 binding site attenuated miR-802-mediated suppression (Figure 8B, 8C). Thus, Sjp40 inhibits miR-802/*Prkaa1* axis through CD36 in human HEPG2 cells.

## Discussion

It is well recognized that the prevalence of helminth infections correlates negatively with the incidence of some metabolic diseases, including obesity 29, and the underlying mechanism(s) whereby these parasites regulate the host metabolism may provide new therapeutic targets for obesity. In this study, we used multiple murine infection models with *S. japonicum* infection or SEA and Sjp40 injection to report, for the first time, that schistosome infection or schistosome-derived molecules can downregulate host miRNA-802 expression, which interacts with mouse *Prkab1* or human* Prkaa1*, resulting in activation of the AMPK pathway and subsequent suppression of hepatic lipogenesis.

Schistosomiasis is recognized as one of the most important of the human parasitic diseases and is characterized by the development of a significant granulomatous inflammatory response, which is induced by schistosome eggs that become entrapped in the liver 30. It is noteworthy that lipogenesis-related indicators, such as elevated serum cholesterol levels and triglyceride and hepatic lipid accumulation, accompanied by the formation of egg-induced granulomas, were significantly decreased in *S. japonicum*-infected mice, especially in those mice fed with high-fat diet. This outcome evoked our strong interests in unveiling the inherent mechanisms in the relationship between helminth infection and host metabolism, further seeking some therapeutics targets to be potentially effective against obesity.

MicroRNAs (miRNAs) not only participate in the regulation of hepatic metabolism, but also serve as novel diagnostic or therapeutic targets for obesity and some metabolic syndromes 31. In the present study, based on miRNA chip data and KEGG pathway analysis, miR-802 was selected and found to be aberrantly decreased in the livers of* S. japonicum* infected mice or in *in vitro* SEA-stimulated hepatocytes. Importantly, our data first established a role for miR-802 in promoting lipogenesis through overexpression and knockdown technology. It has been reported that increased expression of miR-802 is a possible biomarker of type 2 diabetes and disordered glucose metabolism in obese people and mice 32, 33, our study further provide evidence that abnormally expression of miR-802 in obesity plays a causative role in lipid metabolic dysregulation, at least in part, by targeting the hepatic AMPK signaling.

AMPK is a highly conserved master regulator of multiple metabolic pathways and may have therapeutic importance for treating obesity, non-alcoholic fatty liver disease (NAFLD), type 2 diabetes (T2D) 34-36. Liver-specific activation of AMPKα1 decreased lipogenesis and completely protected against hepatic steatosis by reducing hepatic triglyceride accumulation in mice 37. AMPK activators, such as pioglitazone and PF-06409577, were shown to reduce hepatic fat content and improve splanchnic/peripheral glucose uptake in patients with type 2 diabetes and nonalcoholic fatty liver disease (NAFLD) 38, 39. Through loss and gain of function approaches, we found that downregulated miR-802 promoted the expression of mouse *Prkab1* (a regulatory β subunit of AMPK, AMPKβ1) through direct targeting, and the subsequent upregulation of the AMPK pathway. Similarly, human *Prkaa1* (an activated α subunit of AMPK, AMPKα1) but not *Prkab1*, could be functioned by miR-802, and resultantly induced activation of the AMPK pathway in human HepG2 cells. These findings are consistent with earlier studies that *Prkab1*^-/-^ mice have significantly reduced hepatic AMPK activity and acetyl-CoA carboxylase phosphorylation, resulting in reduced rates of hepatic lipogenesis 40, 41. Considering that AMPK activation prevents hepatic lipid accumulation 42, our study revealed a vital role for miR-802 and its target gene, AMPK, as an important axis to participate in the crosstalk between schistosome infection and host metabolic regulation.

Since schistosome eggs lodge in the liver, schistosome-derived molecules might play in altering miR-802 expression and affecting hepatic metabolism. Our studies found that schistosome eggs-derived soluble antigens (SEA) had some certain potent inhibition of hepatic lipid accumulation. More importantly, Sjp40, a major component of schistosome egg-derived antigens was found to exhibit inhibitory effects on the expression of lipogenesis-related genes both in murine, human hepatocytes and hepatic lipid formation in mice via regulating miR-802/AMPK signaling pathway. Some literature addressed that in HFD-fed mice, hepatocyte-specific disruption of CD36, one scavenger receptor involved in fatty acid uptake 43, could improve steatosis by suppressing triglyceride, diacylglycerol, and cholesterol ester content 25. Interesting, Sjp40 could interact with host hepatocytes through the CD36, which suppressed NF-κB signaling-mediated miR-802 expression and finally regulated hepatic lipid metabolism through activating AMPK pathway. In our study, Sjp40 activated AMPK pathway through CD36 on the cell membrane of hepatocytes, but the results showed that Sjp40 treatment inhibited the expression of CD36. It was reported that the NF-κB inhibitor BAY 11-7082 partially abrogated CD36 expression at both the mRNA and protein levels 44. Thus, we speculated that Sjp40 might inhibit CD36 through suppressing the activity of NF-κB. Thus, Sjp40 is considered to represent a potentially novel target for the treatment of obesity-associated fatty liver diseases.

Collectively, our translational research not only establish a new paradigm for the Sjp40 triggered miR-802 reaction which revitalizes AMPK activity and inhibits hepatic lipogenesis (Figure 7K), but also deepens our understanding of the relationship between host metabolism and parasite infection. In addition, Sjp40 and its downstream sensor, miR-802, might warrant further investigation as the potential therapeutic targets for treating obesity-related liver disorders.

## Materials and methods

### Ethics statement

The animal care and use protocols were approved by the Institutional Animal Care and Use Committee (IACUC) of Nanjing Medical University for the use of laboratory animals (Approval Number: 1601004). All experiments were performed in strict accordance with the Regulations for the Administration of Affairs Concerning Experimental Animals.

### Animals, parasites, eggs and antigen preparation

Male C57BL/6 mice (aged 6 weeks) were purchased from the Animal Core Facility of Nanjing Medical University and fed in a specific pathogen-free environment in the Animal Core Facility of Nanjing Medical University. CD36^-/-^ mice on C57BL/6 background were a kind gift from Prof Qiang You (Second Affiliated Hospital, Nanjing Medical University), age (6 weeks old) and gender (male)-matched WT mice with identical genetic backgrounds were used as controls.

*Oncomelania hupensis* infected with the Chinese strain of *S. japonicum*, were purchased from Jiangsu Institute of Parasitic Diseases (Wuxi, China).

*S*. *japonicum* eggs were isolated from the livers of infected C57BL/6 mice as described previously 45. Briefly, soluble egg antigens (SEA) of *S. japonicum* were prepared 46 and the concentration of SEA was assayed using the Bicinchoninic Acid Protein Assay Kit (Pierce, Rockford, IL, USA). The endotoxin concentration of the SEA sample was < 0.03 EU/ml as measured by using a timed gel endotoxin detection kit (Sigma, St. Louis, MO, USA).

HSP40 (Sjp40) plasmid (pET28a-Sjp40) was expressed in *E. coli* BL21 (DE3) cells (Qingke, Beijing, China). The transformed bacteria were grown in LB medium at 37 °C overnight. Growth was monitored by absorbance measurements at 600 nm (OD600). When the OD600 reached 0.6, the expression was induced with 1 mM IPTG at 37 °C for 4 h. In a typical protocol 47, 48, cells were grown in 2L of LB medium and the harvested cells were re-suspended in binding buffer (20 mM Na_2_PO_4_, 0.5 M NaCl, 30 mM imidazole). The suspension was sonicated and then centrifuged at 15,000 rpm for 60 min at 4 °C. The supernatant was collected, and then filtered through 0.45 μm and then 0.22 μm membranes. HisTrap HP (GE, Chicago, USA) and AKTA pure 100 were used for protein automatic purification. Protein purification involved firstly an affinity chromatography step (Ni Sepharose High Performance affinity resin); then the Sjp40 protein was washed using washing buffer (20 mM Na_2_PO_4_, 0.5 M NaCl, 50 mM imidazole). When the system indicates a single peak, we collect the protein in the centrifuge tube. Then the recombinant Sjp40 protein was ultrafiltered and concentrated using a 50 ml 10 kDa ultrafiltration device (Merck Millipore, Billerica, MA, USA). The purity of Sjp40 was examined by SDS-PAGE with Coomassie blue staining and Western Blot. Finally, we stored the protein into -80 °C.

### Experimental mouse models

#### Normal Chow Diet (ND)-*Schistosoma japonicum* Infection Mouse Model

Forty-eight 6-week-old male C57BL/6 mice were separated randomly into two groups and fed a normal diet (ND) containing 10% kcal fat (Research Diets Inc., New Brunswick, NJ, USA). Snails were placed in deionized water and exposed to incandescent light for 3-4 h for cercarial release. For infection, the cercariae were counted and placed on glass cover slips by a 10 μl bacteriological loop 49. Each mouse in the experimental group (ND-inf) was infected with 10 ± 1 *S. japonicum* cercariae via shaved abdomen and the other unchallenged group was used as the normal control (ND-con). Eight randomly selected mice from each group were sacrificed at 3, 6, 9, and 12 weeks post-infection.

#### High Fat Diet (HFD)-Chronic Infection Mouse Model

Twelve 6-week-old male C57BL/6 mice that had been maintained on a high-fat diet (45% kcal fat, 35% kcal carbohydrate, and 20% kcal protein; Research Diets Inc.) for 1 month were randomly divided into two groups: a high-fat diet-chronic infection group (HFD-inf) and the control group (HFD-con). The method of infection was the same as described above, and mice were sacrificed 9 weeks after the infection.

#### Lentivirus Injection Mouse Model

Twenty-four 6-week-old male C57BL/6 mice were randomly divided into four groups: the Lv-Ctrl, Lv-miR802, LV-Ctrl+inf+PZQ, and LV-miR802+inf+PZQ groups. Six-week-old mice in the LV-Ctrl+inf+PZQ and LV-miR802+inf+PZQ groups were infected with 10 ± 1 *S. japonicum* cercariae for 7 weeks and treated with praziquantel (PZQ) by gavage at a dose of 150 mg/kg/day on two consecutive days 23. Mice in the LV-Ctrl and LV-miR802 groups were synchronously fed and these did not receive the schistosome infection or PZQ treatment. After PZQ treatment, each mouse in the four groups was intravenously administered 5×10^7^ TU lentivirus (LV-Ctrl) or lentivirus-miR-802 (LV-miR802) in 200 μl PBS once a week continuously for four weeks. All the mice were sacrificed 7 days after the last injection of the lentivirus.

#### Normal Chow Diet (ND)- or High Fat Diet (HFD)-SEA Treatment Mouse Model

Twenty-four 6-week-old male C57BL/6 mice were kept on HFD or ND for 7 weeks and separated into treatment or controls groups: a normal diet SEA-injected group (ND-SEA), a normal diet saline-injected group (ND-Saline), a high-fat diet SEA-treated group (HFD-SEA) and its control group (HFD-Saline). Mice in the treatment group were injected *i.v*. with 50 μg of SEA in 100 μl saline once a week for 4 weeks. All the mice were sacrificed 7 days after the last injection with SEA.

#### Normal Chow Diet (ND)- or High Fat Diet (HFD)-Sjp40 Treatment Mouse Model

Twenty-four 4-week-old male C57BL/6 mice were kept on HFD or ND for 3 weeks and separated into treatment or controls groups: a normal diet Sjp40-injected group (ND-Sjp40), a normal diet saline-injected group (ND-Saline), a high-fat diet Sjp40-treated group (HFD-Sjp40) and its control group (HFD-Saline). Mice in the treatment group were injected *i.p*. with 50 μg of Sjp40 in 100 μl saline twice a week for 10 weeks. All the mice were sacrificed after the last injection of Sjp40.

### Body weight and food intake

Body weight of mice was determined three times per week from 0 to 9 weeks after infection. Food intake was measured three times per week starting from the time of infection until the mice were sacrificed.

### Blood biochemistry

Total cholesterol (TC), triglyceride (TG), high-density lipoprotein cholesterol (HDL-C), and low-density lipoprotein cholesterol (LDL-C) levels were measured using an Olympus AU5400 automatic biochemical analyzer (Olympus, Tokyo, Japan) 50.

### Histological analysis

Liver samples were fixed with 4% (w/v) paraformaldehyde, embedded in paraffin, cut into 4-μm-thick sections, and stained with hematoxylin and eosin (H&E) for morphological investigations. H&E staining was carried out after deparaffination as previously described 51. Frozen 8-μm-thick sections of liver tissues were stained with Oil Red O (ORO) to detect neutral lipids 52. Cells were washed three times with PBS, fixed with 10% paraformaldehyde for 30 min and then stained with 0.6% Oil Red O for 10min. After hematoxylin staining, cells were observed by microscope (Carl Zeiss, Jena, Germany).

Immunofluorescent staining of liver tissues was performed as we reported 53. Briefly, liver tissues were frozen in optimum cutting temperature compound, cut at a thickness of 5 μm, fixed with acetone and methanol (1:1) for 30 minutes, then ruptured in Triton X-100 for 30 minutes at room temperature. After the non-specific binding sites were blocked with 0.2% BSA for 60 minutes at 4 °C, the sections were incubated with primary antibodies at 4 °C overnight. After washing extensively in PBS, with Alexa Fluor 488- or rhodamine-conjugated secondary antibodies at room temperature for 60 minutes. The tissue sections were then mounted with DAPI, observed under a scanning microscope (Carl Zeiss, Jena, Germany) and analyzed by Image-Pro-Plus 6.0 software (Media Cybernetics, Silver Spring, MD, USA). The following primary antibodies were used for immunofluorescence: mouse anti-albumin (1:200 dilution, Abcam, Cambridge, UK) and rabbit anti-Prkab1 (1:200 dilution, Proteintech Group, Chicago, USA).

### Isolation of murine primary hepatocytes

Murine primary hepatocytes (MPHs) were isolated from wild-type, 8-week-old to 12-week-old C57BL/6 mice or CD36^-/-^ mice by the modified protocol 54. Briefly, mice were anaesthetized, and the liver was perfused with 0.5 mg/ml type Ⅳ collagenase (Gibico, Carlsbad, CA, USA) via the inferior vena cava to isolate murine primary hepatocytes. Purity of live hepatocytes was routinely ≥ 90% by trypan blue exclusion. Cells were seeded for 3 hours on collagen-coated 12-well plates in Williams Medium E (Sigma, St Louis, MO, USA) supplemented with 5% (v/v) fetal bovine serum, 100 units/ml penicillin, and 100 μg/ml streptomycin (4 × 10^5^ cells/well). The medium was replaced after cell attachment. Unless otherwise noted, functional assays and gene expression analysis were performed 24 hours after murine primary hepatocytes plating.

### Cell culture and treatment

The FL83B murine hepatocyte cell line derived in 1969 by Charity Waymouth at the Jackson Laboratory was kindly provided by the Liver Transplantation Center of Jiangsu Province Hospital. HEK293T (human embryonic kidney cells) and HEPG2 (human hepatocellular carcinoma) were purchased from the Cell Bank of the Chinese Academy of Sciences (Shanghai, China). FL83B, HEPG2 and HEK293T cells were cultured on plastic aseptic dishes in DMEM supplemented with 10% (v/v) FBS and penicillin (100 IU/ml)/streptomycin (100 mg/ml). Cells were maintained at 37 °C in a humidified atmosphere containing 5% CO_2_.

FL83B cells were stimulated with A769662 (Selleck, State of Texas, USA, 100 μM) for 12 h, then transfected with the miR-802 mimic or si-*Prkab1* (RiboBio, Guangzhou, China). FL83B cells were stimulated with Compound C (10 μM, MCE, State of New Jersey, USA) for 12 h after transfection with the miR-802 inhibitor or OE-*Prkab1*.

Eggs, SEA (10 μg/ml), and Sjp40 (10 μg/ml) were used to stimulate the murine hepatocyte cell line (FL83B), murine primary hepatocytes, and HEPG2 cell line. For co-culture experiments with* S. japonicum* eggs, cells were treated for up to 3 days with either complete medium alone or complete medium supplemented with purified live eggs (1,000 eggs/ml), which were separated from the cells using a cell culture insert within a 24-well-plate transwell system. Cells were stimulated with 200μM oleic acid and 100μM palmitic acid. Then, cells were collected for RT-PCR analysis to determine the expression level of miR-802, *Prkab1, Prkaa1, Prkaa2*, and lipid metabolism-related genes, and to undertake western blot analysis to detect related proteins.

### Quantitative RT-PCR

Total RNA was extracted from the cells, sera and tissues of the mouse models using RNAiso Plus kits (TaKaRa Biotechnology Co. Ltd., Dalian, China). Total RNA quantity was determined using a NanoDrop Ultramicro-Spectrophotometer (Thermo Fisher Scientific, MA, USA). Reverse transcription of RNA was performed using the PrimeScript RT Master Mix kit (TaKaRa Biotechnology Co. Ltd.). Stem-loop qPCR was used to detect the level of miR-802-5p (RiboBio, Guangzhou, China). Real-time PCR was performed using SYBR Green Master Mix kits (Roche, Basel, Switzerland) and detected by the LightCycler® 96 Real-Time PCR System (Roche). The sequences of primers for miR-802, *Prkab1*, *Prkaa1*, *Prkaa2*, and metabolic indicators are shown in Table S4. The cycling parameters were as follows: stage 1, 50 °C for 2 min and 95 °C for 10 min; stage 2, 40 cycles of 95 °C for 15 s and 60 °C for 1 min; stage 3, 95 °C for 15 s, 60 °C for 30 s and 95 °C for 15 s; and the fold change was calculated using the 2^-△△^Ct method. Relative expression of miR-802, Prkab1, Prkaa1, Prkaa2 and metabolism-related genes were calculated using the 2-ΔΔct method and normalized to β-actin. miR-802 CT values were normalized to U6.

### Western Blotting

Proteins were extracted from the hepatocytes and liver tissues using RIPA lysis buffer (Beyotime, Shanghai, China) with protease and phosphatase inhibitors (Thermo Fisher Scientific). Protein concentration was quantified using the BCA reagent (Thermo Fisher Scientific) following the manufacturer's protocol. An equal amount of protein from each sample was loaded into each lane for separation by SDS-PAGE and then transferred to PVDF membranes (Merck Millipore, Billerica, MA, USA). After blocking with 5% (w/v) skim milk powder dissolved in PBS containing Tween-20 (PBST) at room temperature for 2 hours, the membranes were incubated at 4 °C overnight with the primary antibodies: rabbit anti-Prkab1 (Proteintech Group), rabbit anti-Prkaa1 and Prkaa2 (Cell Signaling Technology, Danvers, MA, USA), rabbit anti-pAMPK (Cell Signaling Technology), rabbit anti-ACC pan (Abcam), rabbit anti-pACC (Abcam), rabbit anti-CD36 (Abcam), rabbit anti-p65 (Abcam), rabbit anti-p-p65 (Cell Signaling Technology) and mouse anti-β-actin (Abcam). After washing with PBST, the membranes were incubated at room temperature for 1 hour with the horseradish peroxidase (HRP) conjugated goat anti-rabbit IgG or goat anti-mouse IgG (Abcam). The density of each band was quantified by densitometric analysis with Image Lab 6.0 software.

### *Prkab1* and *Prkaa1* 3'UTR Luciferase Reporter Vector Construction

The mouse *Prkab1* gene was amplified from the cDNA of mouse liver using the primers (forward) 5'-GGTACCGAGCTCGGATCCATGGGCAACACGAGCAG-3' and (reverse) 5'-GGGTTTAAACGGGCCCTCTAGATCATATCGGCTTGTAGAGGAGGG-3' and inserted into the pcDNA3.1(+) plasmid to generate the recombinant OE-*Prkab1* plasmid. The *Prkab1* 3'UTR and *Prkab1* 3'UTR mut plasmids (forward: 5'- GGACTAGTTGTTGCTTGTTCCAAAAGAAGAGCTC-3', reverse: 5'- CCCAAGCTTCAAGTCAGGGTTTTGAAAACAGTAACAAAAG-3', mutation reverse: 5'- CCCAAGCTTCAAGTCAGGGTTTTGAAAAGACTTAGAAAAG-3') were constructed by amplifying and inserting the 3'UTR and 3'UTR mut of the *Prkab1* mRNA into the pMIR-Report Luciferase vector, respectively. The human *Prkaa1* 3'UTR was amplified by PCR from the human cDNA using the primers (forward) 5'-GGACTAGTATAATGTTCCTGATGTTAACAGAAGACTG-3' and (reverse) 5'-CCCAAGCTTGCAATATTTAAATATTTTCAAAATAAAACACAGTAACTAAAATG-3' and inserted into the pMIR-Report Luciferase vector to generate the luciferase reporter vector. The *Prkaa1* 3'UTR mut plasmids (forward: 5'- GGACTAGTATAATGTTCCTGATGTTAACAGAAGACTG-3', mutation reverse: 5'-CCCAAGCTTGCAATATTTAAATATTTTCAAAATAAAACTCTGAATCTAAAATG-3') were constructed by amplifying and inserting the 3'UTR mut of the *Prkaa1* mRNA into the pMIR-Report Luciferase vector. The concentrations of the extracted plasmid DNAs were measured using a NanoDrop Ultramicro-Spectrophotometer (Thermo Fisher Scientific). The integrity of these constructs was verified by sequencing.

### Dual-luciferase reporter assay system

Twenty-four hours before transfection, 5×10^4^ 293T cells/well were cultured in 24-well plates. Cells were then transfected with 40 nM miR-802 mimic or a negative control and co-transfected with 0.8 μg of wild-type 3' UTR-luc or mutant 3'UTR-luc per well using Lipofectamine 2000 according to the manufacturer's instructions. The pGL-4.74 vector (0.1 μg/well) was co-transfected as the endogenous control for luciferase activity. Luciferase activities were assayed 24 h after transfection using a Dual Luciferase Reporter Gene Assay Kit (Beyotime).

The miR-802 promoter was amplified from mouse genomic DNA template and cloned into the pGL-3-basic vector. A promoter containing a mutation in the NF-κB binding site was also inserted into the empty vector. The luciferase reporter assay was performed as described above 55.

### Transfection

The transfections of miR-802 mimic, NC mimic, miR-802 inhibitor, NC inhibitor, si-*Prkab1*, and OE-*Prkab1* into FL83B cells and murine primary hepatocytes were performed using Lipofectamine 2000 (Invitrogen, Carlsbad, CA, USA) according to the manufacturer's instructions. The miR-802 mimic and its negative control (NC mimic), as well as the miR-802 inhibitor and its negative control (NC inhibitor) were purchased from GenePharma (Shanghai, China). The small interfering RNA targeting *Prkab1* (si-*Prkab1*) was obtained from RiboBio (Guangzhou, China). All the sequences used in the transfections are shown in Table S5. The recombinant OE-*Prkab1* plasmid was re-constructed as described in the following section.

Before transfection, 1×10^5^ cells were plated in a 24-well format with 500 μl DMEM containing 10% FBS without antibiotics. The cells were 90% confluent at the time of transfection. Two μl of Lipofectamine 2000 reagent was added into 50 µl serum-free medium, which mixed with 0.8 µg of DNA and incubated for 20 min, and then the mixture was added into the cells. Cells incubated at 37 °C in CO_2_ incubator for 6 hours. Then the medium would be replaced with DMEM containing 10% FBS. The RNA or protein extraction was 24-48 hours after transfection 56.

### Lentivirus Generation

Lentiviruses were prepared using previously described methods 57, 58. The miRNA (miR-802) expression plasmid was constructed in two steps. First, the precursor stem-loop plus RFP coding sequences of miR-802 were amplified from the pTRIPZ plasmid (Open Biosystems, AL, USA) and then inserted into another lentiviral plasmid, pCDH-CMV-MCSEF1-copGFP (System Bioscience, CA, USA), to create a new modified pCDH lentiviral plasmid (mpCDH containing both GFP and RFP signals. Second, the precursor stem-loop of miR-802 was amplified using the primers (forward) 5'-CAGAAGGCTCGAGAAGGTATATTGCTGTTGACAGTGAGCG-3' and (reverse) 5'-CTAAAGTAGCCCCTTGAATTCCGAGGCAGTAGGCA-3'and cloned into mpCDH to produce a new plasmid, pCDH-miR-802. The 293T cells were co-transfected with the lentiviral plasmid pCDH-miR-802, packaging vector psPAX2 and envelope vector pMD2.G to obtain the recombinant lentivirus. The supernatants were collected 48 h after transfection and concentrated with PEG-8000. LV titers were determined using the end point dilution method by counting the numbers of infected green 293T cells (based on GFP expression) under a fluorescence microscope at 96 h after infection. The titer in IU ml^-1^ = (the numbers of green fluorescent cells) × (dilution factor)/(volume of virus solution).

### Acetyl-CoA carboxylase activity

FL83B cells were collected in an extraction solution from the ACC Activity Assay Kit (Solarbio, Beijing, China). After ultrasonication, the cell suspension was centrifuged at 8,000 g for 10 min. Levels of ACC activity were determined by incubating the suspension with Molybdenum blue from the ACC Activity Assay Kit (Solarbio).

### Hepatocyte lipogenesis assay

Cultured murine primary hepatocytes in 12-well plates were washed with PBS and incubated in 250 μl reaction buffer (10% BSA, ^3^H-glucose, Williams Medium E). The cells were incubated for 4 h at 37 °C and then washed twice with cold PBS before adding 200 μl HCl each well. The cells were incubated at room temperature for 10 min, transferred to EP tubes and the cells ruptured thoroughly by vortexing. Two μl of the cell suspension was taken to determine the protein concentration by BCA reagent. Total cellar lipids were extracted from the cell suspension using 800 μl chloroform/methanol (2/1, v/v), vortexed and centrifuged. For lipogenesis assay, organic phase radioactivity was measured by liquid scintillation counting. To detect fatty acid synthesis, the organic phase evaporated to dry at room temperature overnight in tube with screw and dissolved by 50 μl hexane. Then incubated in 100 °C for 30 min with 75 μl 5% H_2_SO_4_ methanol solution and 125 μl methanol. Above solution dissolved in 125 μl ddH_2_O and extracted by petroleum. Petroleum phase combined in scintillation vial with 3 ml scintillation liquid for radioactivity assay 59.

### miRNA expression microarray

RNAs from 5 combined liver samples from each ND-con and ND-inf groups (10 ± 1 *S. japonicum* cercariae, 9 weeks post infection) were isolated using RNAiso Plus kits (TaKaRa Biotechnology Co. Ltd.) following the manufacturer's protocol. After assessing the RNA integrity and concentration on a NanoDrop Ultramicro-Spectrophotometer (Thermo Fisher Scientific), the samples were labeled using the miRNA Complete Labeling and Hyb Kit (Agilent technologies, Santa Clara, CA, US) followed the manufacturer's instructions, labeling section. Each slide was hybridized with 100ng Cy3-labeled RNA using miRNA Complete Labeling and Hyb Kit (Agilent technologies) in hybridization Oven (Agilent technologies) at 55 ℃, 20 rpm for 20 hours according to the manufacturer's instructions, hybridization section. After hybridization, slides were washed in staining dishes (Thermo Shandon, Waltham, MA, US) with Gene Expression Wash Buffer Kit (Agilent technologies). Slides were scanned by Agilent Microarray Scanner (Agilent technologies) and Feature Extraction software 10.7 (Agilent technologies) with default settings. Raw data were normalized by Quantile algorithm, Gene Spring Software 11.0 (Agilent technologies). The results (Figure 1A and Table S1) have been deposited in the Database of Genotypes and Phenotypes (dbGAP) of the National Center for Biotechnology Information (United States National Library of Medicine, Bethesda, MD, USA) under accession number GSE134866.

### Pierce pull-Down polyhistidine assay

To validate the interactions between Sjp40 and CD36, His tagged Sjp40 proteins were purified as previously described. FL83B cells lysed in Pierce Lysis Buffer supplemented with 0.5 mM PMSF (Sigma). The His pull-down was performed by a Pierce Pull-Down Polyhistidine Assay kit following a previously published protocol 60. Protein separation and detection were performed using SDS-PAGE. Antibodies against the following proteins were used: anti-Sjp40 and anti-CD36.

### Statistical analyses

All analyses were conducted with SPSS software (version 19.0; SPSS Inc, Chicago). Differences in significance between two groups were determined using the Student's *t*-test. Multiple comparisons were performed by one-way ANOVA, and followed by LSD post-test for comparison between two groups. Values are presented as the mean ± s.e.m. *P* values <0.05 were considered as statistically significant; significant differences are presented as follows: * *P*<0.05, ** *P*<0.01, *** *P*<0.001.

## Supplementary Material

Supplementary figures and tables.

## Abbreviations

AMPK: AMP-activated protein kinase
ACC: acetyl-CoA carboxylase
CHOL: cholesterol
Cpt1: carnitine palmitoyl transferase 1
Dgat: diacylglycerol acyl coenzyme A acyltransferase
FA: fatty acid
Fas: fatty acid synthase
HDL-C: high-density lipoprotein cholesterol
HFD: high-fat diet
HSP: heat shock protein
LDL-C: low-density lipoprotein cholesterol
LV: lentivirus
Mcd: malonyl coenzyme A decarboxylase
MPHs: murine primary hepatocytes
NAFLD: non-alcoholic fatty liver disease
ND: normal diet
NF-κB: nuclear factor-kappa B
OA: oleic acid
ORO: oil red O
PA: palmitic acid
Ppp2ca: protein phosphatase 2 catalytic subunit alpha
Pafah1b1: platelet activating factor acetylhydrolase 1b regulatory subunit 1
PZQ: praziquantel
SEA: soluble egg antigens
*S. japonicum*: *Schistosoma japonicum*
Scd1: stearoyl-CoA desaturase-1
Srebp1c: sterol regulatory element binding protein 1C
T2D: type 2 diabetes
TG: triglyceride
Ucp2: uncoupling protein 2
