# Supplementary material for: Therapeutic inhibition of miR-802 protects against obesity through AMPK-mediated regulation of hepatic lipid metabolism
Source: Theranostics. 2021 Jan 1;11(3):1079–99. doi: 10.7150/thno.49354 (PMC7738900; doi:10.7150/thno.49354)
Supplement: Supplementary file 1 — Supplementary figures and tables. [file thnov11p1079s1.pdf]

Supporting Information

Therapeutic inhibition of miR-802 protects against obesity through AMPK-mediated regulation of hepatic lipid metabolism

Yangyue Ni [†], Zhipeng Xu [†], Chen Li [†], Yuxiao Zhu, Ran Liu, Fan Zhang, Hao Chang, Maining Li, Liang Sheng, Zhong Li, Min Hou, Lin Chen, Hong You, Donald P. McManus, Wei Hu, Yinong Duan, Yu Liu, Minjun Ji [*]

[†] Yangyue Ni, Zhipeng Xu and Chen Li contributed equally to this work.

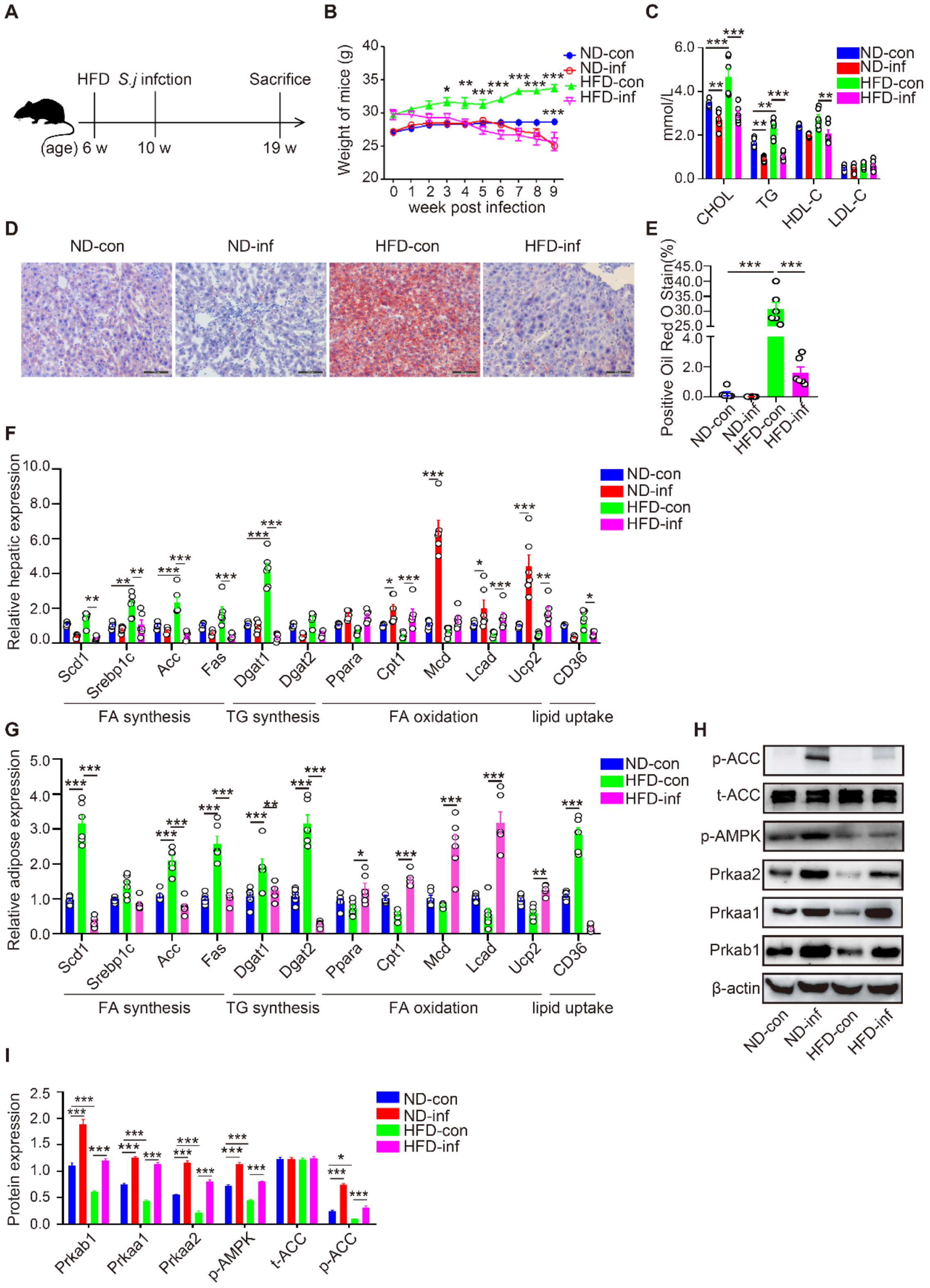

Figure S1. *Schistosoma japonicum* infection improves lipid metabolism in HFD mice.

(**A**) Sixteen 10-week-old male C57BL/6 mice were separated equally into two groups and fed a normal diet. Each mouse in the ND-inf group was infected with 10 ± 1 *S.*

japonicum cercariae and the ND-con group was established as the normal control group. Sixteen 6-week-old male C57BL/6 mice that had been maintained on a high-fat diet for 1 month were randomly divided into two groups: a high-fat diet-chronic infection group (HFD-inf) and the control group (HFD-con). Mice were sacrificed 9 weeks after the infection. (**B**) Dynamic changes in body weight of ND- and HFD-fed mice after infection with *Schistosoma japonicum*. (**C**) Cholesterol, TG, HDL-C, LDL-C levels in sera of HFD mice upon infection. (**D**, **E**) Representative images of liver sections from ND-con, HFD-con, ND-inf or HFD-inf mice stained with Oil red O. (**F**) qRT-PCR quantification of lipid-related genes expression in livers of four groups of mice (ND-con, HFD-con, ND-inf or HFD-inf). (**G**) qRT-PCR quantification of lipid-related gene expression in adipose tissues of three groups of mice (ND-con, HFD-con and HFD-inf). Data are expressed as the mean ± s.e.m. of three independent experiments with 6 mice per group in each experiment. (**H**, **I**) Detection of Prkab1, Prkaa1, Prkaa2, phosphorylated AMPK, total ACC and phosphorylated ACC levels in the livers of ND- or HFD-infected mice. Data are expressed as the mean ± s.e.m. for each group, and are representative of one typical experiment out of three; * P <0.05, ** P <0.01, *** P <0.001.

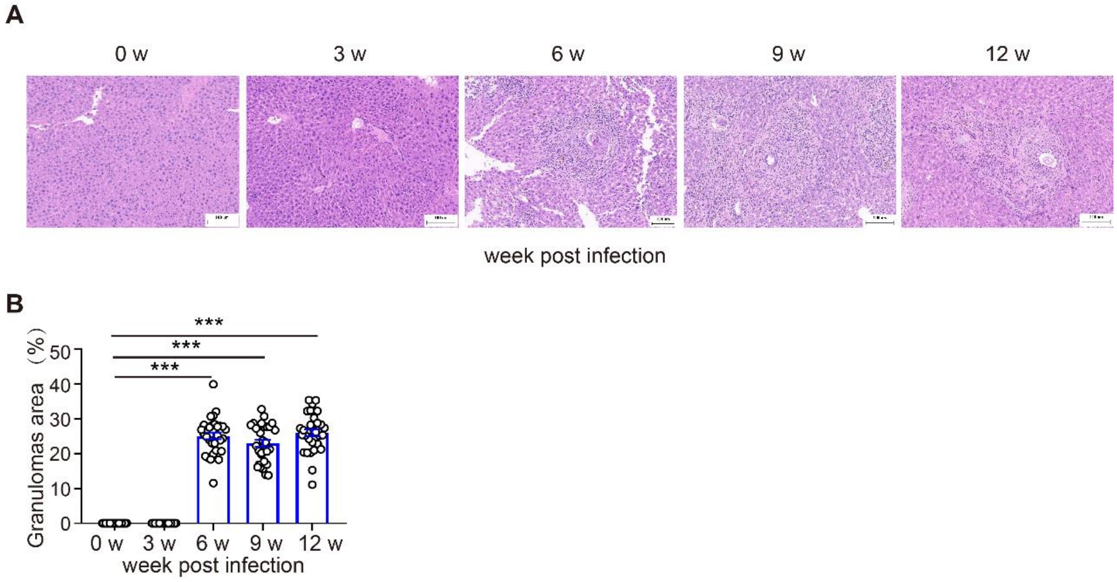

Figure S2. Pathological changes in the liver of mice infected with *Schistosoma japonicum*.

(**A**, **B**) H&E staining of murine liver sections at 0w, 3w, 6w, 9w and 12w after *S. japonicum* infection, scar bar, 100 μm. Data are expressed as the mean ± s.e.m. of 6 mice for each group in one representative experiment. For each mouse, the sizes of 5 liver granulomas around single eggs were quantified. All experiments were repeated twice, * *P* <0.05, ** *P* <0.01, *** *P* <0.001.

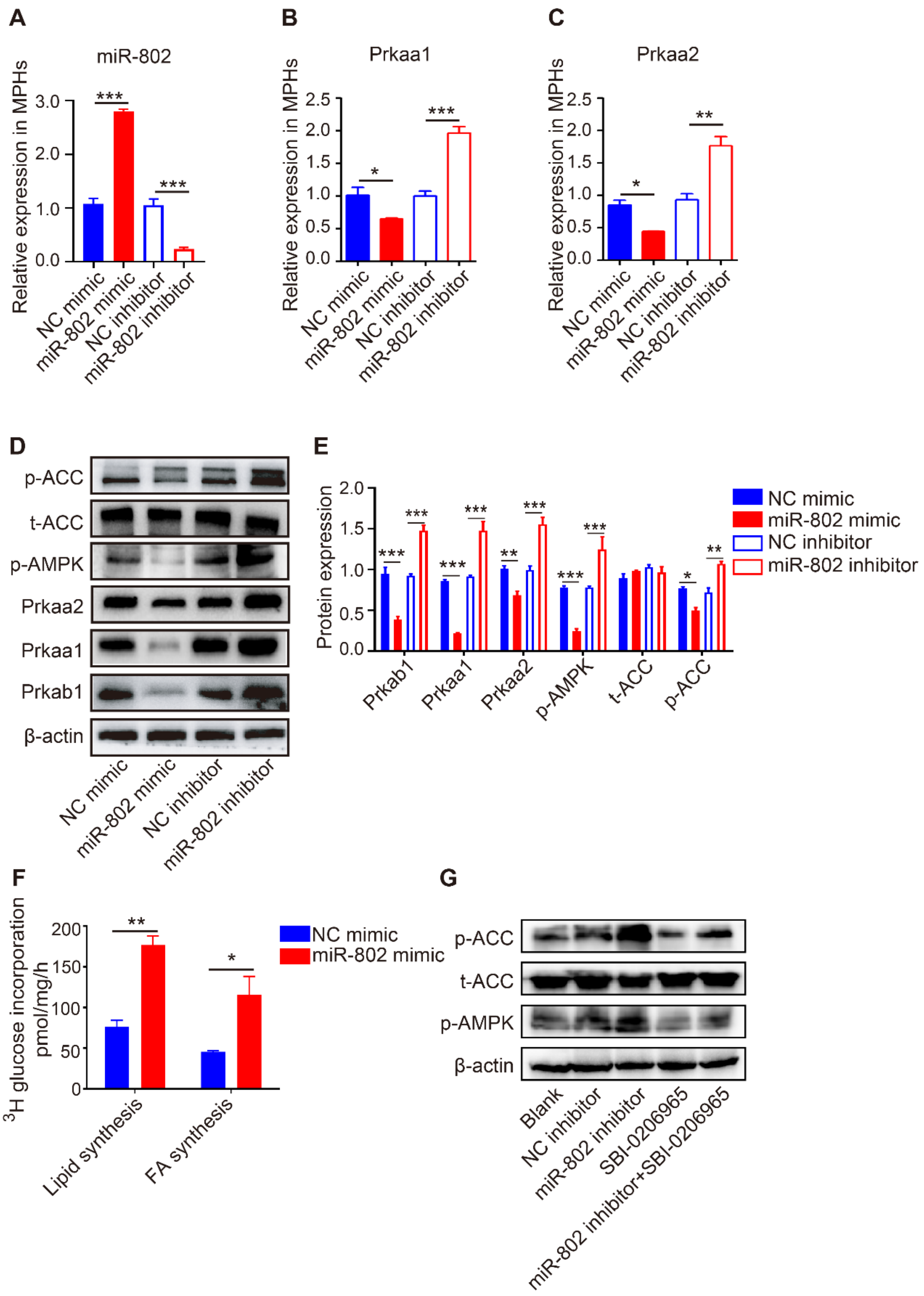

Figure S3. miR-802 decreases AMPK expression in murine primary hepatocytes.

(**A**-**C**) qRT-PCR analysis of miR-802, *Prkaa1* and *Prkaa2* expression in MPHs upon transfection with miR-802 mimic or inhibitor. (**D, E**) Western blot analysis of Prkab1,

Prkaa1, Prkaa2, p-AMPK, t-ACC and p-ACC in MPHs after stimulating with miR-802 mimic or inhibitor. (**F**) ^{3}H-glucose-derived lipid or FA in MPHs in the presence of miR-802 mimic. (**G**) . Western blot analysis of phosphorylated AMPK and ACC in FL83B cells after stimulating with miR-802 inhibitor, SBI-0206965 or miR-802 inhibitor + SBI-0206965.Data are expressed as the mean ± s.e.m of 3 independent experiments with similar results. *P <0.05, **P <0.01, ***P <0.001.

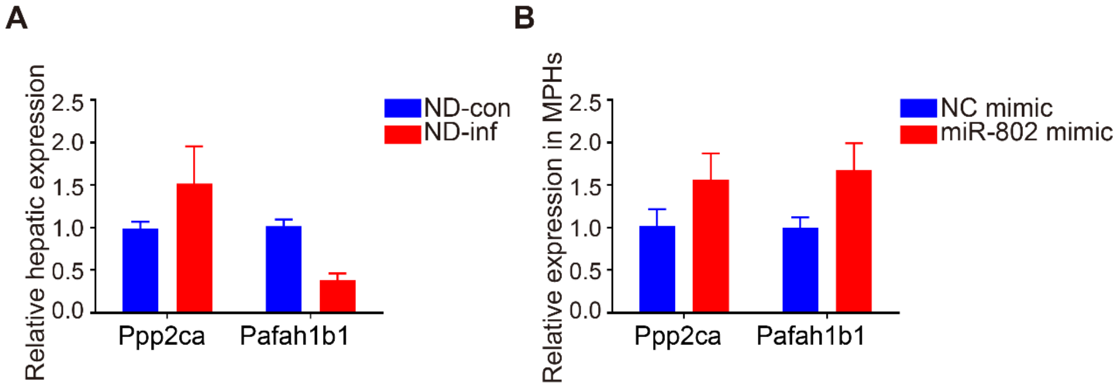

Figure S4. The expression of Ppp2ca and Pafah1b1, other predicted target genes of miR-802, associated with metabolism.

(**A**) qRT-PCR quantification of *Ppp2ca*, and *Pafah1b1* in the liver of mice in the ND-con and ND-inf groups. (**B**) qRT-PCR quantification of *Ppp2ca*, and *Pafah1b1* in MPHs after treating with miR-802 mimic. Error bars represented mean ± s.e.m. of three independent repeats.

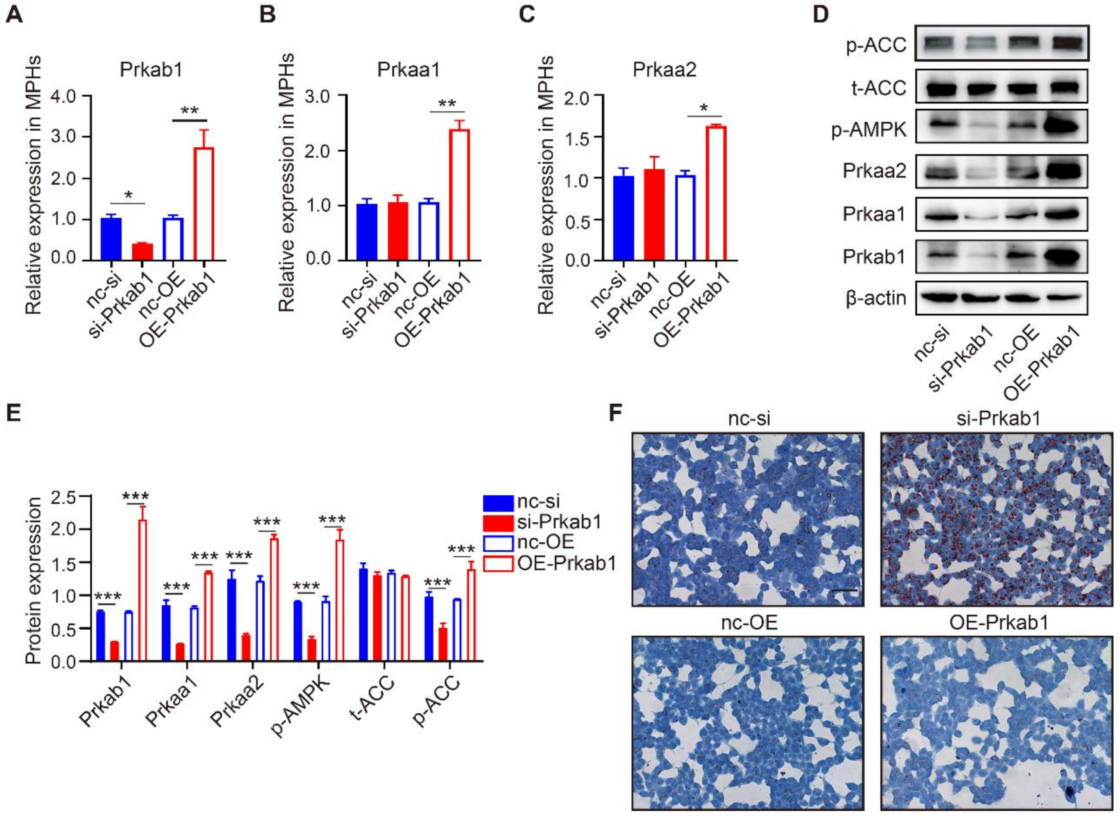

Figure S5. Prkab1 enhances the level of Prkaa1 and Prkaa2 in murine primary hepatocytes.

(**A**-**C**) qRT-PCR analysis of *Prkab1*, *Prkaa1* and *Prkaa2* expression in MPHs upon transfection with si-*Prkab1* or OE-*Prkab1*. (**D, E**) Western blot analysis of Prkab1, Prkaa1, Prkaa2, p-AMPK, t-ACC and p-ACC in MPHs cells following stimulation with si-*Prkab1* or OE-*Prkab1*. (**F**) Oil red O staining after treatment with OE-*Prkab1* and si-*Prkab1*.Data are expressed as the mean ± s.e.m of 3 independent experiments with similar results. *P <0.05, **P <0.01, ***P <0.001.

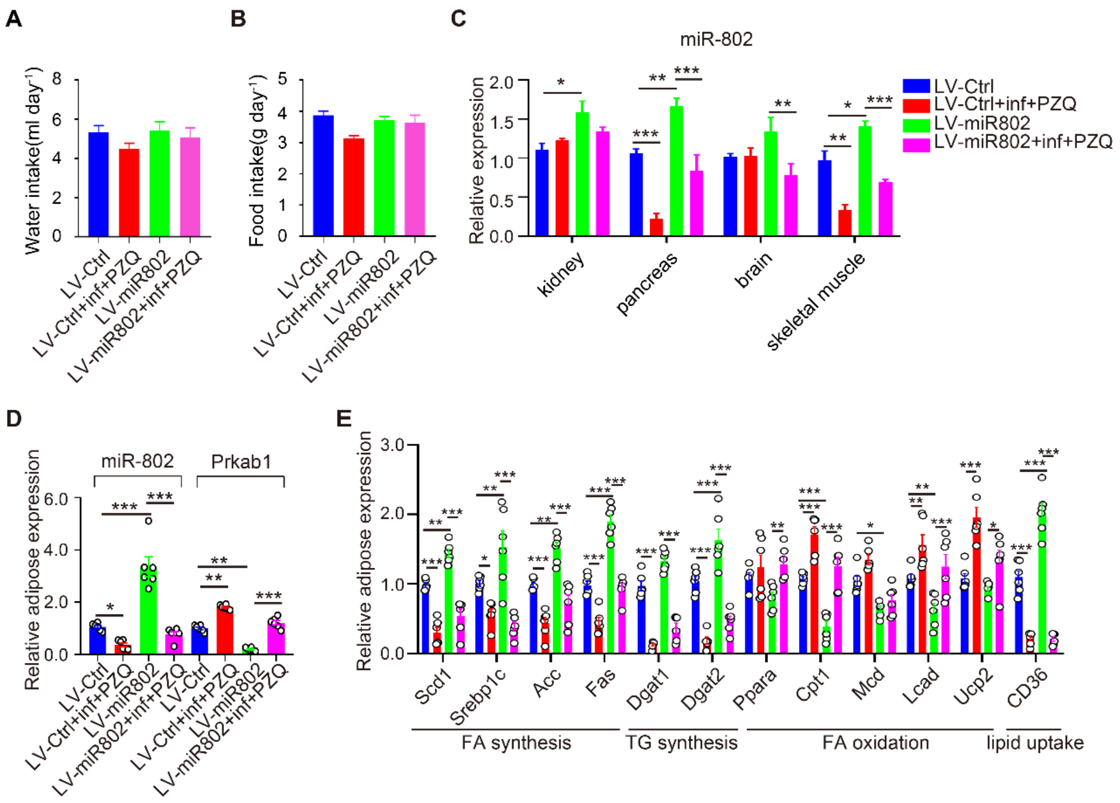

Figure S6. Overexpression of miR-802 up-regulates lipogenesis related genes in adipose tissue.

(**A, B**) Water intake and food intake of mice in the LV-Ctrl, LV-miR802, LV-Ctrl+inf+PZQ, and LV-miR802+inf+PZQ groups. (**C**) qRT-PCR quantification of miR-802 in kidney, pancreas, brain and skeletal muscle. (**D**) qRT-PCR quantification of miR-802 and *Prkab1* in adipose tissue. (**E**) qRT-PCR quantification of lipogenesis related genes in adipose tissue. Data are expressed as the mean ± s.e.m. of 6 mice for each group in one representative experiment. All experiments were repeated twice, **P* <0.05, ***P* <0.01, ****P* <0.001.

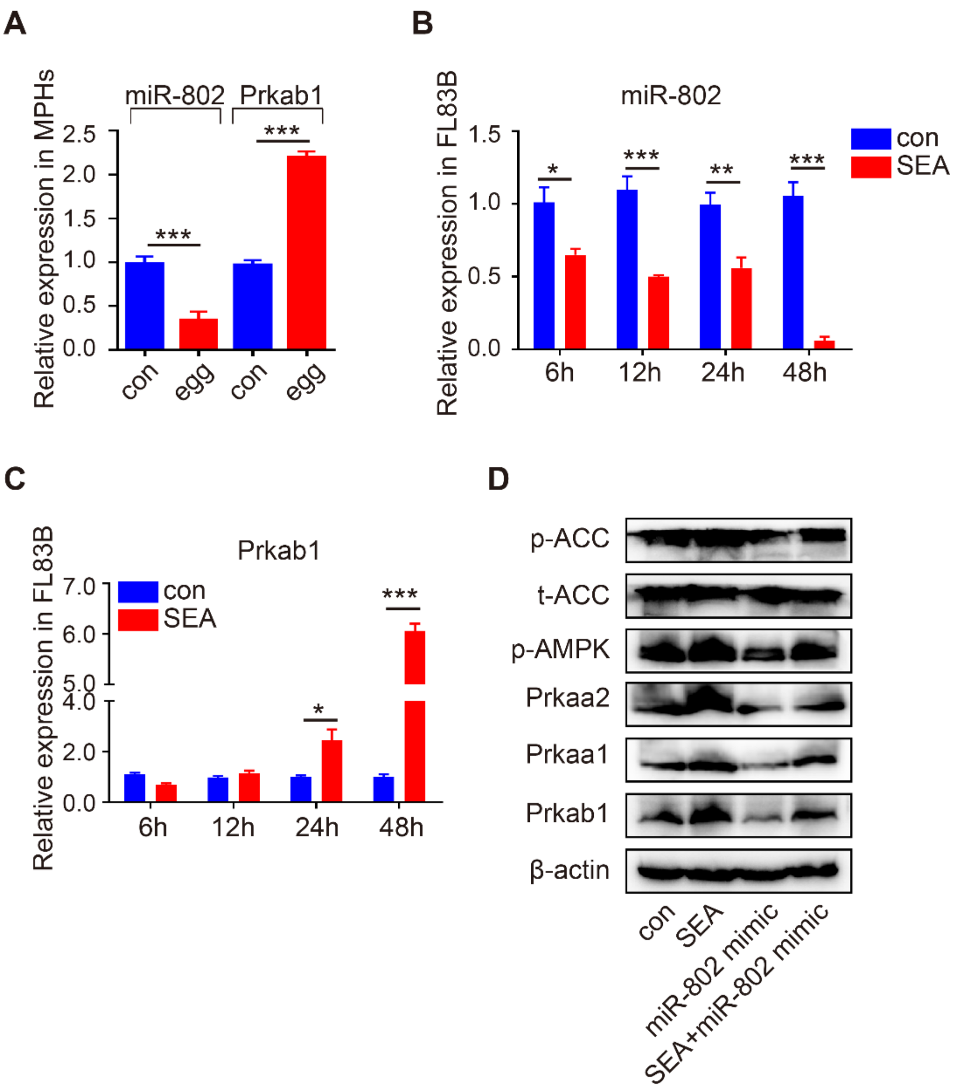

Figure S7. SEA stimulation suppresses the expression of miR-802 in murine hepatocytes.

(**A**) *S. japonicum* eggs (1 × 10^3) were incubated in complete DMEM medium and placed in the upper part of transwell chamber, separated from lower chamber placed with 2.5 × 10^5 MPHs. Then, qRT-PCR quantification of miR-802 and *Prkab1* in MPHs after co-culturing with eggs of *S. japonicum* after 72h. (**B**, **C**) qRT-PCR quantification of miR-802 and *Prkab1* in FL83B cells stimulated with 10 µg/ml of SEA for 6 hours, 12 hours, 24 hours, and 48 hours. (**D**) Rescue effects of mir-802 mimic in FL83B cells. Error bars: mean ± s.e.m. of three independent repeats. *P <0.05, **P <0.01, ***P <0.001.

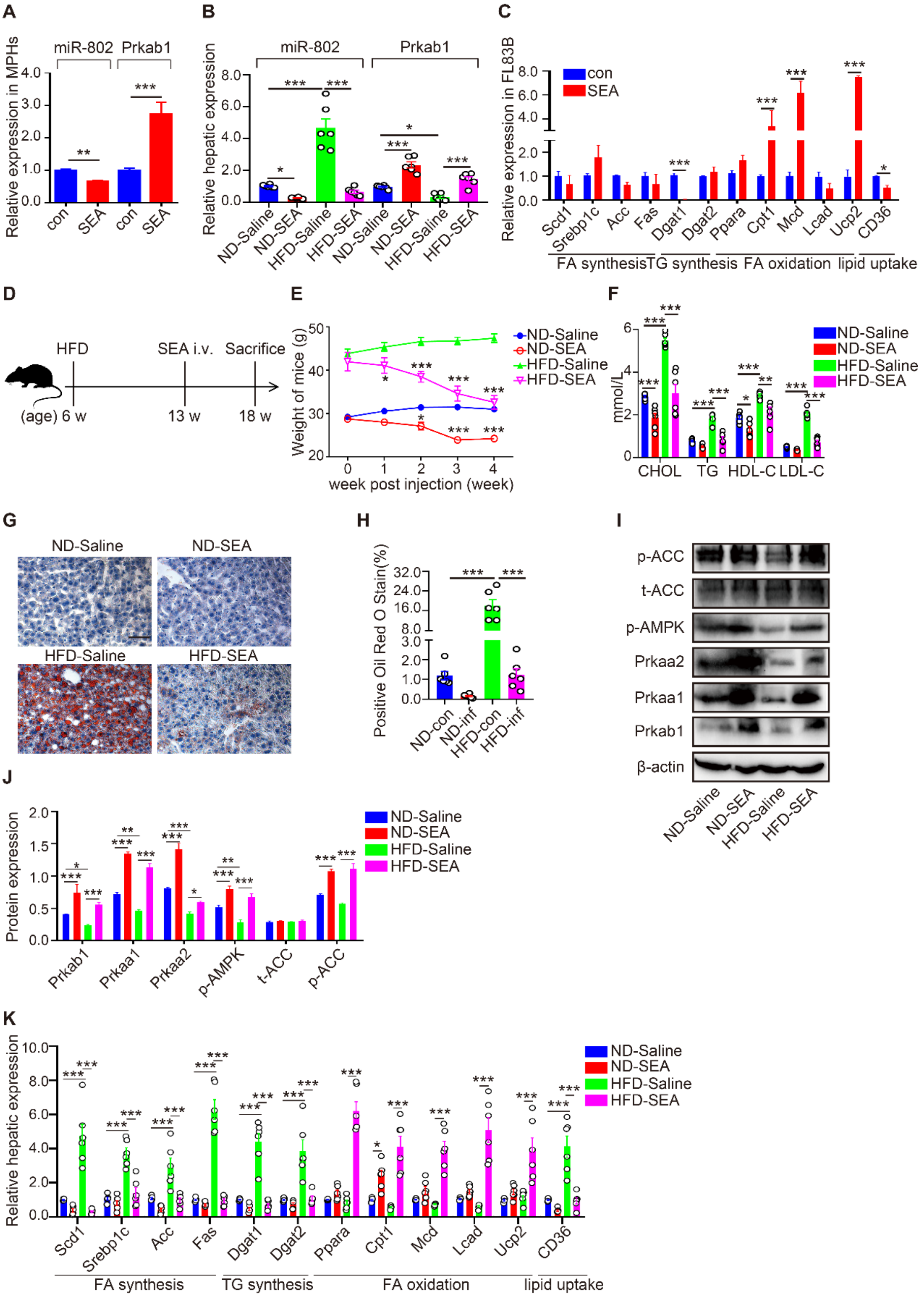

Figure S8. Soluble egg antigen of *S. japonicum* improves host lipid metabolism in HFD mice by down-regulating miR-802.

(**A**) qRT-PCR analysis of the expression of miR-802 and *Prkab1* in MPHs treated with SEA for 24 hours. (**B**) The expression of miR-802 and *Prkab1* in hepatic tissues from

ND-Saline, ND-SEA, HFD-Saline and HFD-SEA groups was determined by qRT-PCR. (**C**) qRT-PCR analysis of lipogenesis-related genes in FL83B cells upon SEA treatment for 48 hours. Data are expressed as the mean ± s.e.m of 3 independent repeats. (**D**) Mice were kept on a high fat diet or normal diet were injected *i.v.* with 50 μg of SEA in 100 μl Saline once a week for 4 weeks. (**E**) Dynamic changes in body weight of ND- and HFD-fed mice after injection with SEA. (**F**) Cholesterol, TG, HDL-C, LDL-C levels in sera of HFD mice upon treatment with SEA. (**G**, **H**) Representative images of liver sections from ND-Saline, HFD-Saline, ND-SEA or HFD-SEA mice stained with Oil red. (**I**, **J**) Levels Prkab1, Prkaa1, Prkaa2, phosphorylated AMPK, total ACC, phosphorylated ACC in the livers of four groups of mice. Data are expressed as the mean ± s.e.m. for each group, and are representative of one typical experiment out of three. (**K**) qRT-PCR quantification of lipid-related genes expression in livers of four groups of mice. Data are expressed as the mean ± s.e.m of two repeated experiments, n = 6. *P <0.05, **P < 0.01, ***P < 0.001.

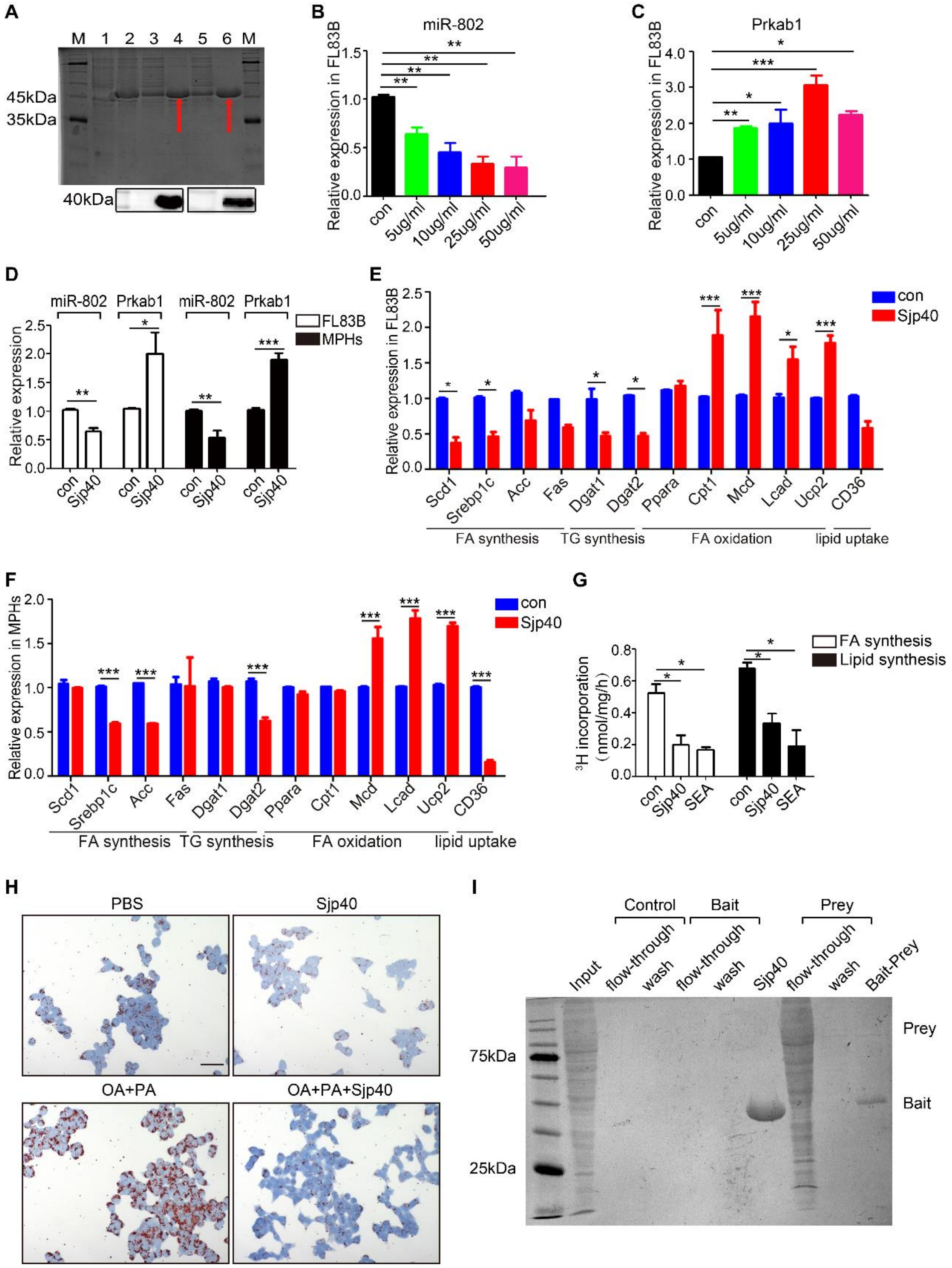

Figure S9. Sjp40 stimulation improves lipid metabolism in murine hepatocytes by down-regulating the expression of miR-802.

(**A**) Identification of Sjp40 expression (1. Ultrasound-disrupted *E. coli* cells transformed with pET28a plasmid, 2. Ultrasound-disrupted *E. coli* cells transformed with pET28a-Sjp40 plasmid, 3. Supernatant of ultrasound-disrupted *E. coli* cells

transformed with pET28a-Sjp40 plasmid, 4. Precipitate of ultrasound-disrupted *E. coli* cells transformed with pET28a-Sjp40 plasmid, 5. Supernatant of *E. coli* cells transformed with pET28a-Sjp40 plasmid before affinity chromatography, 6. Sjp40 protein after purification). (**B**, **C**) qRT-PCR quantification of miR-802 and *Prkab1* in FL83B cells stimulated with Sjp40 at 5, 10, 25, 50 μg/ml. (**D**) qRT-PCR quantification of miR-802 and *Prkab1* upon treating with Sjp40 (10 μg/ml) in FL83B cells and primary hepatocytes for 24 h. (**E**) qRT-PCR quantification of lipogenesis-related genes expression in FL83B cells after stimulating with Sjp40 (10 μg/ml) for 24 h. (**F**) qRT-PCR quantification of lipogenesis-related genes expression in primary hepatocytes of mice after stimulating with Sjp40 (10 μg/ml) for 24 h. (**G**) ^{3}H-glucose-derived lipid or FA in primary hepatocytes in the presence of Sjp40 (10 μg/ml) for 24 h. (**H**) Sjp40 led to a significant decrease in lipid content in FL83B cells as displayed by Oil Red staining. Error bars of *in vitro* experiments: mean ± s.e.m. of three independent repeats. **P* <0.05, ***P* <0.01, ****P* <0.001. (**I**) All samples from pull-down assay were separated and analyzed by 12% SDS-PAGE. Group control is the negative control without Sjp40.

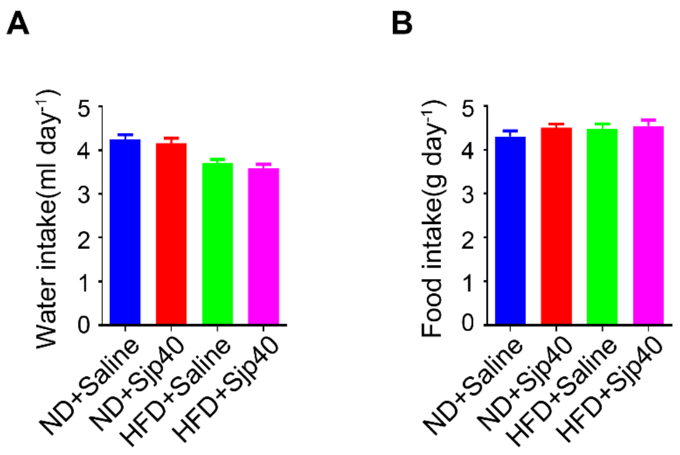

Figure S10. Sjp40 had no effect on diet and drinking water.

(**A, B**) Water intake and food intake of ND-Saline, ND-Sjp40, HFD-Saline and HFD-Sjp40 mice. All error bars indicate mean ± s.e.m of 4 mice from each group in one representative experiment.

Table S1. Top 10 up-regulated and down-regulated miRNAs in the liver miRNA microarray from normal and *S. japonicum*-infected mice.

Top 10 up-regulated miRNAs (**A**) and 10 down-regulated miRNAs (**B**) in the liver miRNA microarray chip from 5 mixed liver samples in each ND-con and ND-inf groups were selected.

A. Top 10 up-regulated miRNAs

Systematic name	Uninfected	Chronic infection
mmu-miR-146b-5p	0	6.680273
mmu-miR-1907	0	6.022519
mmu-miR-3061-5p	0	5.799051
mmu-miR-1967	0	5.592492
mmu-miR-130b-3p	0	5.490145
mmu-miR-432	0	5.410701
mmu-miR-1931	0	5.302338
mmu-miR-3110-3p	0	5.249473
mmu-miR-296-5p	0	5.164032
mmu-miR-5113	0	5.104465

B. Top 10 down-regulated miRNAs

Systematic name	Uninfected	Chronic infection
mmu-miR-487b-3p	0	-5.84202
mmu-miR-3057-5p	0	-5.23264
mmu-miR-802-5p	0	-4.87403
mmu-miR-7a-1-3p	0	-4.70159

mmu-miR-192-3p	0	-4.6003
mmu-miR-31-3p	0	-4.35632
mmu-miR-219-5p	0	-4.06588
mmu-miR-676-3p	0	-3.9274
mmu-miR-744-5p	0	-3.83358
mmu-miR-3962	0	-3.78822

Table S2. Prkab1 related pathways

Term	Input number	Background number	*P*-Value
AMPK signaling pathway	2	129	0.008151
Oxytocin signaling pathway	2	158	0.011949
Circadian rhythm	1	31	0.032557
Longevity regulating pathway - multiple species	1	64	0.065048
Adipocytokine signaling pathway	1	73	0.073723
Hypertrophic cardiomyopathy (HCM)	1	84	0.084219
Longevity regulating pathway	1	96	0.095537
Glucagon signaling pathway	1	102	0.101145
Insulin resistance	1	111	0.109493

Table S3. Prediction of combination of Sjp40 and CD36.

Name	Predicted value of Delta G (binding free energy)	Predicted value of Kd (dissociation constant)
Schistosoma japonicum isolate Hunan major egg antigen mRNA (Sjp40)	-14.27	3.45e-11

Table S4. Primer sequences for qRT-PCR.

Gene	Forward (5’-3’)	Reverse (5’-3’)
miR-802[†]	ACACTCCAGCTGGGTCAGTA ACAAAGATTC	TGGTGTCGTGGAGTCG
prkab1[†]	AGTCTACTTGTCTGGGTCCT T	GCTGGCTGGTTACTATTGG
prkaa1[†]	GCGTGTACGAAGGAAGAAT	CGAGGGAGGTGACAGATGA
prkaa2[†]	TCCAGGCTTGAAACCACAT	AGACCTCTGCTCCACCACC
scd1[†]	CTCTACACCTGCCTCTTCGG	GCCGTGCCTTGTAAGTTCTG
srebp1c[†]	CAGAGCCGTGGTGAGAAGC	GCAAGAAGCGGATGTAGTC G
acc[†]	CACCAGTTTTGCATTGAGAA C	TACGCTGTTGAGTTCATAGG C
fas[†]	AGGTGGTGATAGCCGGTATG T	TGGGTAATCCATAGAGCCCA G
dgat1[†]	GTTTCCGTCCAGGGTGGTAG T	TGGCACCTCAGATCCCAGTA G
dgat2[†]	GCCTGGGTGCCTTCTGTAA	AGTCTATGGTGTCTCGGTTG A
ppara[†]	ACAAGTGCCTGTCTGTCGG	TCAGGTAGGCTTCGTGGAT
cpt1[†]	GTGTCCAAGTATCTGGCAGT C	TCAGGGTATTTCTCAAAGTC AA
mcd[†]	GGGGCTGTGATGTGGCGTAT	GGGCTACCAGGCTGAGGAT
lcad[†]	AGCCTCCACTCAGATATTGT CA	TGGCGTTCGTTCTTACTCCTT GT

ucp2[†]	GCTGGTGGTGGTCGGAGAT	TTACGGGCAACATTGGGAG
cd36[†]	TGGTCAAGCCAGCTAGAAA	TCCCAAGTAAGGCCATCTC
ppp2ca[†]	TGGGAGACTATGTGGACAGAGGAT	GACAGACCACCGTGTAGACAGAAG
pafah1b1[†]	TTCGTTCAAATGGCTATGAAGAGG	CCAAGAGGACCACCCGACGTAAAT
prkaa1[#]	TTGAAACCTGAAAATGTCCTGCT	GGTGAGCCACAACTTGTTCTT
prkaa2[#]	CTGTAAGCATGGACGGGTTGA	AAATCGGCTATCTTGGCATTCA

[†] genes from mice.

[#] genes from human.

Table S5. Primer sequences for mimic, inhibitor of miR-802 and siRNA of Prkab1.

**Gene**	**Forward** (5'-3')	**Reverse** (5'-3')
NC mimic	UUCUCCGAACGUGUCACG UTT	ACGUGACACGUUCGGAGA ATT
miR-802 mimic†	UCAGUAACAAAGAUUCAU CCUU	GGAUGAAUCUUUGUUACU GAUU
NC inhibitor	CAGUACUUUUGUGUAGUA CAA	
miR-802 inhibitor†	AAGGAUGAAUCUUUGUUA CUGA	
miR-802 mimic#	CAGUAACAAAGAUUCAUC CUUGU	AAGGAUGAAUCUUUGUUA CUGUU
si-*Prkab1*†	CCAGGAGCCTTACATGTCT	

† genes from mice.

# genes from human.
